# Chromosome-specific potential intron polymorphism markers for large-scale genotyping applications in pomegranate

**DOI:** 10.3389/fpls.2022.943959

**Published:** 2022-08-30

**Authors:** Prakash Goudappa Patil, Shivani Jamma, Manjunatha N, Abhishek Bohra, Somnath Pokhare, Karuppannan Dhinesh Babu, Ashutosh A. Murkute, Rajiv A. Marathe

**Affiliations:** ^1^ICAR-National Research Centre on Pomegranate (NRCP), Solapur, India; ^2^State Agricultural Biotechnology Centre, Centre for Crop and Food Innovation, Food Futures Institute, Murdoch University, Murdoch, WA, Australia; ^3^ICAR-Central Citrus Research Institute (CCIR), Nagpur, India

**Keywords:** chromosome, genome, potential intron polymorphism markers, pomegranate, diversity

## Abstract

Despite the availability of whole genome assemblies, the identification and utilization of gene-based marker systems has been limited in pomegranate. In the present study, we performed a genome-wide survey of intron length (IL) markers in the 36,524 annotated genes of the Tunisia genome. We identified and designed a total of 8,812 potential intron polymorphism (PIP) markers specific to 3,445 (13.40%) gene models that span 8 Tunisia chromosomes. The ePCR validation of all these PIP markers on the Tunisia genome revealed single-locus amplification for 1,233 (14%) markers corresponding to 958 (27.80%) genes. The markers yielding single amplicons were then mapped onto Tunisia chromosomes to develop a saturated linkage map. The functional categorization of 958 genes revealed them to be a part of the nucleus and the cytoplasm having protein binding and catalytic activity, and these genes are mainly involved in the metabolic process, including photosynthesis. Further, through ePCR, 1,233 PIP markers were assayed on multiple genomes, which resulted in the identification of 886 polymorphic markers with an average PIC value of 0.62. *In silico* comparative mapping based on physically mapped PIP markers indicates a higher synteny of Tunisia with the Dabenzi and Taishanhong genomes (>98%) in comparison with the AG2017 genome (95%). We then performed experimental validation of a subset of 100 PIP primers on eight pomegranate genotypes and identified 76 polymorphic markers, with 15 having PIC values ≥0.50. We demonstrated the potential utility of the developed markers by analyzing the genetic diversity of 31 pomegranate genotypes using 24 PIP markers. This study reports for the first time large-scale development of gene-based and chromosome-specific PIP markers, which would serve as a rich marker resource for genetic variation studies, functional gene discovery, and genomics-assisted breeding of pomegranate.

## Introduction

Pomegranate (*Punica granatum* L.) is one of the oldest edible fruit crops in the world and is thought to have originated in Iran. It is mainly grown in drier parts of Southeast Asia, Iran, China, Japan, the West Indies, the United States (California), Tropical America, and India (Holland and Bar-Ya'akov, 2014). With respect to taxonomic classifications, pomegranate was placed under the family Lythraceae that includes the genus *Punica* with three species: *Punica protopunica, Punica nana* and *Punica granatum* L. (2n = 16), of which *P. granatum* is cultivated for fruit production (Moriguchi et al., 1987; Graham and Graham, 2014; Berger et al., 2016). Due to its multifaceted health benefits to humankind, pomegranate cultivation has gained wider popularity across the Mediterranean and Middle Eastern countries (Melgarejo et al., 2009; Teixeira da Silva et al., 2013). India, on the other hand, leads the world in pomegranate cultivation, with a total area of 2.83 lakh hectares and a production of 31.83 lakh million tonnes (http://agricoop.gov.in2019-20). The progress of pomegranate research and breeding has remained slow because of the paucity of genomic information in this crop (Saminathan et al., 2016). Recently, the international efforts on genome sequencing of pomegranate have leveraged the genomic repertoire of pomegranate. The availability of genome sequence paves the way for large-scale development of functional DNA markers, i.e., EST-SSRs, EST-SNPs (Ono et al., 2011; Ophir et al., 2014), and miRNA-SSRs (Patil et al., 2020b) in pomegranate.

The availability of genic SSR and SNP markers mined from whole genome assemblies has facilitated high-throughput genetic analysis in various crops. The limitations that hamper the widespread use of these gene-based DNA markers include lower polymorphic potential, as well as the need for specialized and high-cost platforms for marker genotyping. So far, SSR markers have shown to be highly effective in pomegranate genetic analyses, including genetic diversity, population structure, and marker trait association studies (Curro et al., 2010; Pirseyedi et al., 2010; Singh et al., 2015). SSR markers in these studies showed a relatively low degree of DNA polymorphism; as a result, highly polymorphic chromosome-specific markers have recently been developed in pomegranate (Patil et al., 2021).

There are very few reports available on the mapping of gene(s)/QTL for fruit quality traits in pomegranate using SSR and SNP markers. Basaki et al. (2011) identified 14 SSRs significantly associated with 14 traits, explaining 2 to 29% phenotypic variance (PVs) for flower and fruit quality traits in pomegranate. Singh et al. (2015) also reported 4 SSRs significantly associated with fruit weight, titratable acidity, and bacterial blight severity in pomegranate. Harel-Beja et al. (2015), through QTL analysis, identified 25 QTLs for fruit quality traits using SNP markers. Recently, using SNP markers, Trainin et al. (2021) fine mapped the candidate gene, i.e., anthocyanidin reductase (*ANR*), with point mutation being responsible for the black peel color in pomegranate.

Therefore, the development of new gene-based DNA marker systems in pomegranate could greatly support future genomics research and genetic improvement (Patil et al., 2020a,c). Enhanced breeding efficiency through the deployment of DNA markers would accelerate the progress of the cultivar development in pomegranate. Given this, efficient gene-based marker systems with abundant distribution in the genome and the ability to demonstrate polymorphism on simple genotyping platforms are urgently required (Badoni et al., 2016).

Introns, abundant in most eukaryotic genomes, are found in several gene sequence components. Low purifying selection pressure during evolution has caused these introns to remain less conserved and variable than coding regions. These regions can serve as highly polymorphic genetic markers (Badoni et al., 2016). Despite being based on genic regions, these markers have been reported to show greater plant intra-species variations than other types of markers (Muthamilarasan et al., 2014).

The popularity of intron length polymorphism (ILP) is growing because it not only offers similar benefits to SSR but also shows certain unique qualities, including direct representation of variation within specific genes and subspecies (Wang et al., 2006). Similar to SSRs, when primers were designed in flanking exons to amplify introns by PCR, cross-species amplification became possible (Yang et al., 2007).

Huang et al. (2010) developed ILP markers following a comparative genomics approach to determine the positions of introns in the genome. Yang et al. (2007) developed a database of potential intron polymorphism (PIP) markers based on intron position predictions across species. PIP markers have been developed in various plant species (Wang et al., 2010; Chen et al., 2011; Liu et al., 2012), but not in pomegranate yet. The PIP markers could be used in combination with SSR markers to determine genetic diversity given the tremendous advantages they offer in terms of subspecies specificity, neutrality (no phenotypic effect), and the ability to perform assay variation within genes (Huang et al., 2013).

The growing information on structurally and functionally annotated genes made available from whole genome sequencing of many crops is a great resource for the development of ILP markers on a genome-wide scale. Still, there are limited reports on the development of ILP markers in fruit trees in comparison to other DNA marker systems. Earlier, Xia et al. (2017) developed genome-wide markers (SSR, ILP, and PIP) from 16 sequenced tree species. In pomegranate, no research on ILP markers has been reported so far despite the availability of whole genome sequences of four genotypes. Realizing the importance of ILPs in pomegranate research, we carried out the current research with the following objectives: (i) large-scale development of ILP markers based on annotated genes in the Tunisia genome, (ii) to develop a physical map and comparative mapping using four sequenced pomegranate genomes, and (iii) to demonstrate ILP marker application in genetic studies.

## Materials and methods

### Mining and designing of potential intron polymorphism markers

**Figure 1:**
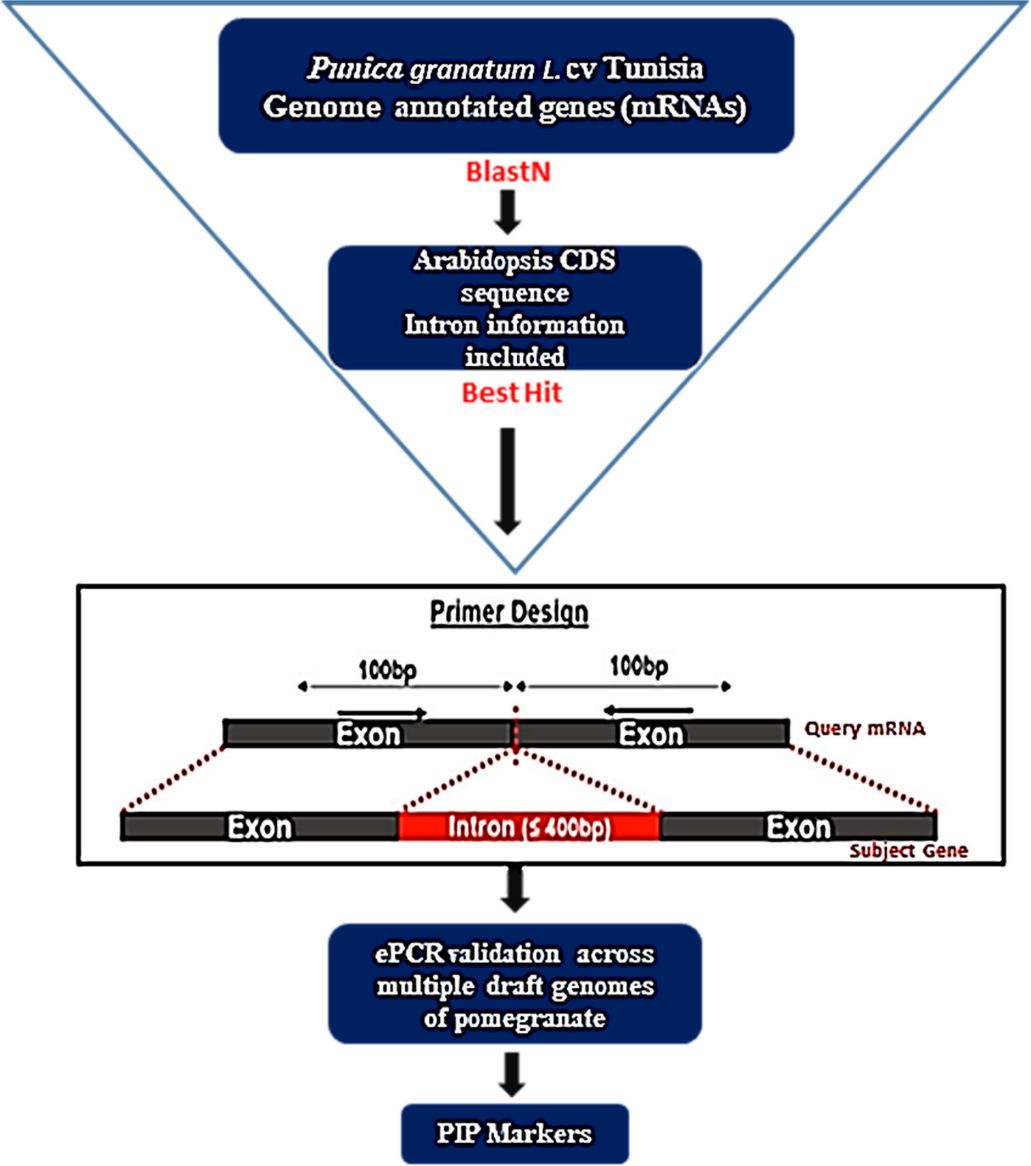
A flowchart depicting the development of PIP marker in pomegranate using the PIP database (Image source: http://ibi.zju.edu.cn/pgl/pip/methodology.html).

To develop intron-specific PIP markers in pomegranate, we retrieved 36,524 annotated gene models (Accession No: XM_031515503.1 to XM_31552026.1, https://www.ncbi.nlm.nih.gov/nuccore?linkname=bioproject_nuccore_transcript&from_uid=580467) for the Tunisia genome (Luo et al., 2020) from the NCBI (https://www.ncbi.nlm.nih.gov/). Complexity reduction was performed to identify 25,710 unique sequences through the CD-HIT-EST tool with default parameters (Li and Godzik, 2006). Using these sequences as a query, we searched against Arabidopsis CDS sequences with introns as a reference to anticipate intron positions in its mRNA sequences using the PIP database (http://ibi.zju.edu.cn/pgl/pip/, Yang et al., 2007) and designed a pair of primers on both sides of each intron position (Figure 1). PIP identifies exon–intron boundaries and predicts suitable primers flanking intronic regions. The identified intron flanking primers were designated as *Pg*_*PIP* (*Punica granatum* potential intron polymorphism).

### Mapping and validation of PIP markers through ePCR

The high-quality “Tunisia” pomegranate genome assembly with eight pseudo-chromosome molecules (Luo et al., 2020) and three other draft genome sequences *cv*. Dabenzi (Qin et al., 2017), Taishanhong (Yuan et al., 2018), and AG2017 (Akparov et al., 2017), were retrieved from the NCBI database. *In silico* simulated PCR was performed by an ePCR algorithm (Schuler, 1997) for all designed PIP markers against eight Tunisia chromosomes using default parameters of GMATA software (Genome-wide Microsatellite Analyzing Tool, Wang and Wang, 2016). As a result, the output file (.emap) was generated with detailed information on the amplification patterns of the markers with approximate amplicon sizes and target positions on Tunisia chromosomes. Then, the PIP markers amplifying a single locus on Tunisia were mapped across the other three pomegranate genomes in order to obtain approximate amplicon sizes to calculate various marker parameters using GenAlEx v. 6.5 (Peakall and Smouse, 2012) software.

### Physical mapping of PIP markers on Tunisia chromosomes

The ePCR provided a preliminary idea about the amplification of all PIP loci with the start and end positions within genes on each Tunisia chromosome. Using the ShinyCircos software (Yu et al., 2017), a circular graph is drawn to illustrate the chromosome-wise localization of each gene and its PIP markers. Apart from that, the saturated PIP marker-based physical map of each chromosome was constructed using MapChart v 2.2 software (Voorrips, 2002), based on the physical positions of all the PIP loci with single amplicons in Tunisia. The functional annotations details of each gene harboring PIP markers were retrieved from the Tunisia genome annotation file (NCBI).

### ePCR validation of PIP markers across genomes

To evaluate the amplification specificity and polymorphism nature of newly designed PIP markers. The single-locus PIP primers identified on the ‘Tunisia' chromosomes were validated against the three draft genome sequences of pomegranate cv. Dabenzi, Taishanhong and AG2017. Using the GMATA software, we identified the approximate product sizes obtained for PIP markers across the four pomegranate genomes. We then used GenAlEx v. 6.5 software to compute various marker parameters: number of alleles (Na), effective alleles (Ne), major allelic frequency (MF), observed (*Ho*) and expected (*He*) heterozygosity, Shannon's information index (*I*), and the polymorphism information content (PIC). The Arabidopsis homolog genes of pomegranate having single PIP marker amplicons were further annotated based on bulk download at GO-TAIR (https://www.arabidopsis.org/tools/bulk/go/index.jsp) to obtain the Gene Ontology Annotation Plot showing three classes: biological process (BP), molecular function (MF), and cellular component (CC).

### PCR-based validation of PIP markers

Using the modified CTAB method, as described by Ravishankar et al. (2000), genomic DNA was extracted from the leaf samples of 31 pomegranate genotypes as listed in Table 1. For the wet lab assay, initially, one hundred PIP markers were synthesized, which were evenly distributed across eight chromosomes of the Tunisia genome. PCR screening was done on a subset of eight pomegranate genotypes, including “Ganesh”, “Arakta”, “P-16”, “Gulesha Red”, “Tabesta”, IC318790, IC1205, and IC318723, using the Prime-96TM Thermal Cycler (HiMedia, India). Based on the results of clear amplifications, subsequently, 24 informative PIP markers were selected for screening of 31 pomegranate genotypes for the genetic diversity study. All the PCR experiments were carried out in 10 μl reaction volume, which included 10 ng of template DNA, 10× PCR buffer, 1 mM dNTP mix, and 10 pmols each of forward and reverse primers, as well as 0.1 U of *Taq* DNA polymerase (Himedia, India). PCR with a touchdown program was followed (Patil et al., 2021), and finally, all the amplicons were resolved and photographed on 3% metaphor gels using a gel documentation system (Vilber Lourmat, France).

**Table 1:** The details of 31 different genotypes of pomegranate used in this study.

**Sl. no**.	**Genotype name**	**Type**	**Origin/source**
1	Bhagawa	Indigenous variety	Maharashtra (India)
2	Ganesh	Indigenous variety	Maharashtra (India)
3	Mridula	Indigenous variety	Maharashtra (India)
4	G-137	Indigenous variety	Maharashtra (India)
5	P-26	Indigenous cultivar	Maharashtra (India)
6	P-23	Indigenous cultivar	Maharashtra (India)
7	P-16	Indigenous cultivar	Maharashtra (India)
8	Co-White	Indigenous cultivar	Tamil Nadu (India)
9	Yercaud Local	Indigenous variety	Tamil Nadu (India)
10	Jodhpur Collection	Indigenous cultivar	Rajasthan (India)
11	Jallore Seedless	Indigenous variety	Rajasthan (India)
12	Surat Anar	Indigenous cultivar	Gujarat (India)
13	Patna-5	Indigenous cultivar	Maharashtra (India)
14	Bassein Seedless	Indigenous cultivar	Karnataka (India)
15	KRS	Local collection	Karnataka (India)
16	Kandhari	Exotic breeding line	Afghanistan
17	GR Pink	Exotic cultivar	Russia
18	Shirin Anar	Exotic cultivar	Russia
19	Spin Saccharin	Exotic cultivar	Maharashtra (India)
20	Nimali	Exotic variety	Srilanka
21	Muscat	Exotic cultivar	Oman
22	Daru-17	Wild collection	Himachal Pradesh (India)
23	IC318703	Wild collection	Himachal Pradesh (India)
24	IC318753	Wild collection	Himachal Pradesh (India)
25	IC318754	Wild collection	Himachal Pradesh (India)
26	IC318779	Wild collection	Himachal Pradesh (India)
27	IC318790	Wild collection	Himachal Pradesh (India)
28	IC318705	Wild collection	Himachal Pradesh (India)
29	IC318723	Wild collection	Himachal Pradesh (India)
30	IC1182	Wild collection	Uttaranchal (India)
31	IC1205	Wild collection	Uttaranchal (India)

### Genetic diversity analysis

The allele sizes (bp) of 24 PIP primers were scored on 31 genotypes. The marker genotyping data were used to compute various marker parameters using GenAlEx v. 6.5 software. DARwin v. 6.0.13 (Perrier and Jacquemoud-Collet, 2006) software was used to create an NJ tree based on pairwise genetic distances determined by using Jaccard's dissimilarity coefficients with 10,000 bootstrap iterations. The same software was also used for Principal coordinate analysis (PCoA).

## Results

### Genome-wide designing of PIP markers

**Figure 2:**
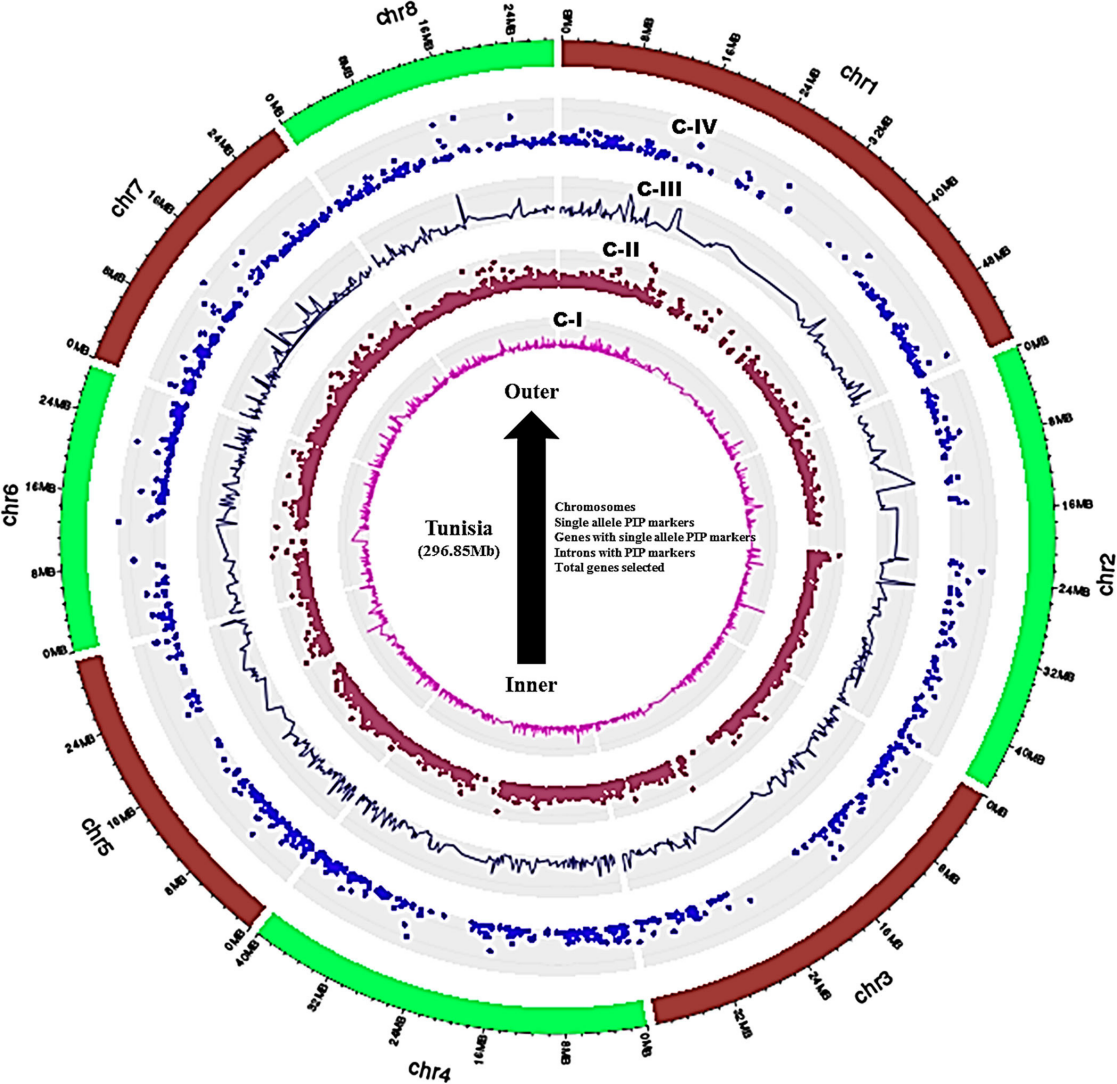
Circos graph depicting the physical positions of genes, intron numbers, and lengths targeted.

A set of 36,524 annotated gene sequences of the Tunisia genome produced 25,710 nonredundant sequences, which were used to design the intron polymorphism based PIP markers by using Arabidopsis as a reference genome in the PIP database. As a result, 8,812 PIP markers were designed out of 25,710 unique gene sequences with an average marker density of 30.76 markers/Mb of the genome. These markers targeted 3,445 (13.40%) gene models that span 8 chromosomes of the Tunisia genome (Supplementary Table 1). The marker density ranged from 22.84 per Mb (Chm_ 1) to 36.60 per Mb (Chm_4 & 6) with an average of 30.76 per Mb. The highest average marker density (36.60/Mb) was recorded for chromosomes 4 and 6, followed by 35.54/Mb and 34.55/Mb for chromosomes 7 and 8, and the lowest (22.84/Mb) was found in chromosome 1 (Table 2). Further, ePCR mapping of 8,812 PIP markers on Tunisia chromosomes revealed that 7,425 (84.26%) markers were successfully validated. Distribution and frequency analysis of physically mapped PIP markers revealed that markers mapped on chromosome 4 (1,317 markers, 89.7%) were more frequent than those mapped on chromosome 2 (707, 67.05%). Out of 7,425, 1,233 (16.60%) markers produced single-locus amplification in Tunisia chromosomes corresponding to 958 (27.80%) genes. Further, the overall distribution of PIP markers on 8 Tunisia chromosomes, their positions, intron numbers, and lengths within 3,445 to 958 chosen genes were reduced from the inside to the outer rings of the Circos graph (Figure 2, Supplementary Table 2).

**Table 2:** ePCR based validation of Pg_PIP markers for genotyping applications.

				**ePCR validation of PIP markers**																			
				**Allele no** **Tunisia genome**					**Allele no** **Dabenzi genome**					**Allele no** **Taishanhong genome**					**Allele no** **AG2017 genome**				
	**Size (Mb)**	**PIP primers**	**Physical density (markers/Mb)**	**1**	**2**	**3**	**>3**	**Total**	**1**	**2**	**3**	**>3**	**Total**	**1**	**2**	**3**	**>3**	**Total**	**1**	**2**	**3**	**>3**	**Total**
Chm_1	55.56	1269	22.84	170	460	441	3	1074	159	509	437	13	1118	165	498	446	4	1113	170	466	438	7	1081
Chm_2	44.57	1056	23.69	120	281	305	1	707	160	387	394	4	945	150	391	400	1	942	156	380	374	1	911
Chm_3	39.96	1035	25.90	157	371	378	0	906	148	376	380	4	908	159	381	369	3	912	136	378	368	0	882
Chm_4	40.13	1469	36.60	234	593	482	8	1317	229	569	494	10	1302	227	569	500	8	1304	217	569	472	9	1267
Chm_5	31.53	956	30.32	137	361	324	6	828	128	361	331	12	832	141	357	324	10	832	135	337	324	6	802
Chm_6	28.33	1037	36.60	142	397	354	3	896	155	376	369	4	904	152	389	363	1	905	160	367	343	6	876
Chm_7	28.78	1023	35.54	163	378	342	6	889	156	364	375	10	905	164	365	369	6	904	145	360	370	5	880
Chm_8	27.99	967	34.55	110	349	346	3	808	129	341	347	4	821	125	347	338	5	815	115	347	328	2	792
Total	296.85	8812	30.76	1233	3190	2972	30	7425	1264	3283	3127	61	7735	1283	3297	3109	38	7727	1234	3204	3017	36	7491

### High-density PIP-marker based physical map

**Figure 3:**
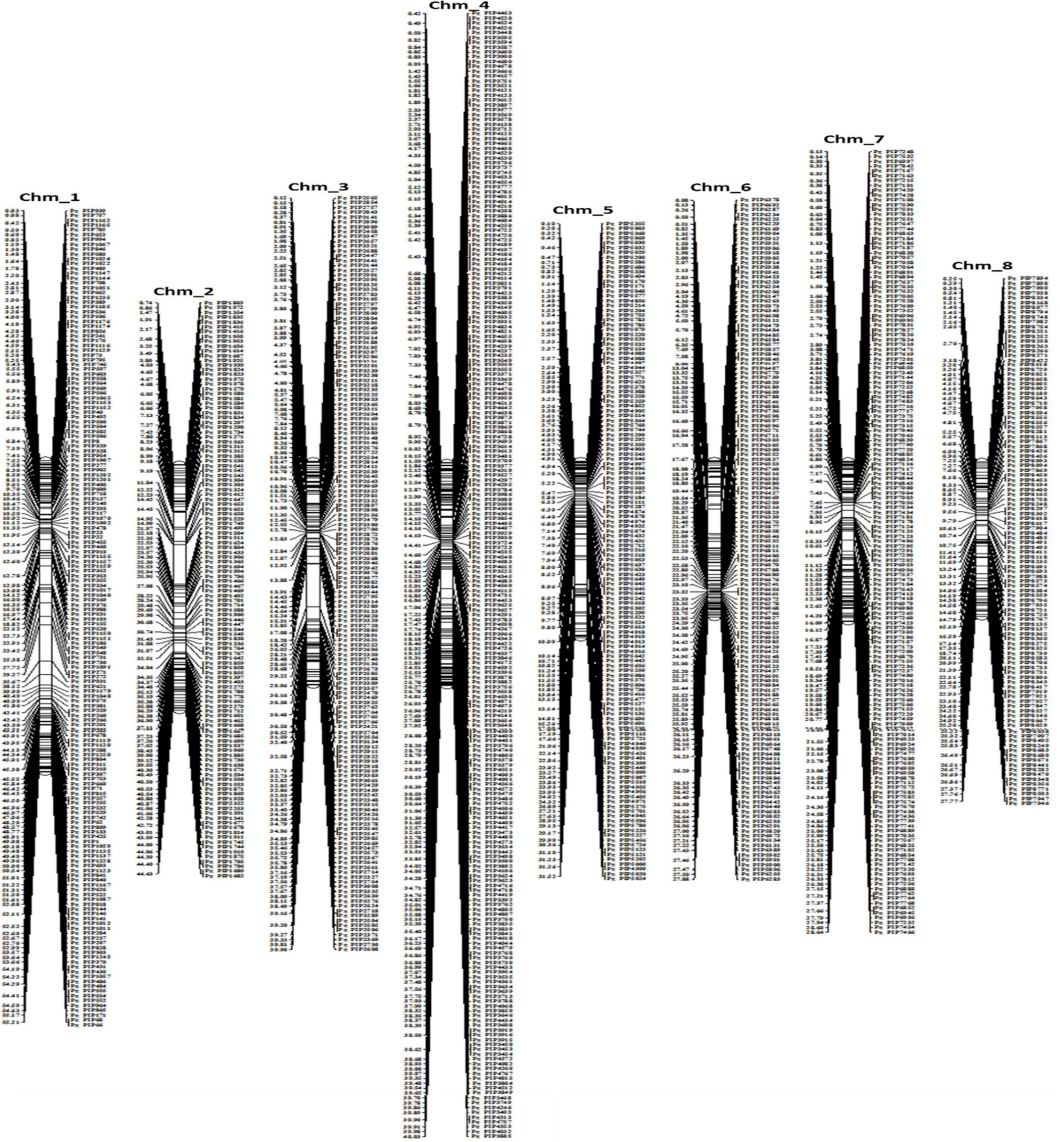
Physical linkage map based on 1233 PIP markers of the Tunisia genome.

On each chromosome, the physical start positions of 1,233 Pg_PIP markers were used to create a high-density physical map (Figure 3, Supplementary Table 3). The map revealed that Chm_4 had the highest number of markers (234), followed by Chm_1 (170), Chm_7 (163), and Chm_3 (157). Whereas Chm_8 had the lowest number of markers (110), followed by Chm_2 (120), Chm_5 (137), and Chm_6 (142).

### ePCR validation of PIP markers in four genomes

Amplification efficiency and specificity of the developed ILP markers were determined by e-mapping 8,812 PIP markers on the “Tunisia” genome. A total of 7,425 (84.26%) primers were mapped and proportionately all the primers got validated across 8 chromosomes of “Tunisia” yielding one to more than 3 alleles. A total of 1,233 (14%) primers produced a single amplicon of the expected size, while 3,190 (36.2%) primers produced two alleles, and 2,972 (33.73%) primers produced three alleles. More than 3 alleles were recorded for 30 (0.34%) primers. Subsequently, validation of 8,812 PIP markers was also performed on three genome assemblies (“Dabenzi,” “Taishanhong,” “AG2017,”). Interestingly, we found, as compared to the Tunisia genome, maximum PIP markers were validated on Dabenzi (7,735, 87.78%) and Tiashanhong (7,727, 87.69%), followed by AG2017 (7,491, 85%). Similarly, a total of 1,283 (“Taishanhong”), 1,264 (“Dabenzi”), and 1,234 (“AG2017”) PIP markers offered single-locus amplifications.

We selected a set of 1,233 chromosome-specific PIP markers that had a single amplicon on the Tunisian genome and validated them in three additional pomegranate genomes (Table 2, Supplementary Table 4). The 886 (71.86%) resultant polymorphic PIP markers generated a total of 429 alleles across the four genomes. The average “Na” per locus was 2.79, ranging from 2 to 5. MF per locus varied from 0.25 to 0.87, with 0.55 being the average. The markers showed PIC values in the range of 0.25 to 1.00, with an average of 0.62. Interestingly, 1,118 PIP markers had PIC values ≥0.50. The average “*I*” index for the four genomes studied was 1.01.

We made chromosome-wise comparisons based on marker parameters (Table 3, Supplementary Table 5). Chm_4 had the most polymorphic markers (160) and the highest average value of Na (637). However, Chm_8 had the least values for both polymorphic loci (71) and Na (300). Whereas, with respect to all other parameters, i.e., Ne, I, *Ho, He*, and PIC, all the chromosomes except Chm_5 showed higher average values. The markers belonging to Chm_2 and Chm_3 had higher average PIC values of 0.67 and 0.66, respectively.

**Table 3:** ePCR-based marker statistics for 1233 chromosome-specific PIP primers assayed on four pomegranate genomes.

**Chromosome**	**TP**	**TNP**	**Na**	**MF**	**Ne**	***I***	***Ho***	***He***	**PIC**
Chm_1	170	118	454 (2.67)	0.53	2.33	0.87	0.94	0.55	0.63
Chm_2	120	101	354 (2.95)	0.52	2.47	0.96	0.96	0.58	0.67
Chm_3	157	130	475 (3.03)	0.53	2.47	0.97	0.94	0.58	0.66
Chm_4	234	160	637(2.72)	0.56	2.30	0.85	0.87	0.52	0.60
Chm_5	137	82	352 (2.57)	0.58	2.20	0.79	0.83	0.49	0.56
Chm_6	142	100	389 (2.74)	0.55	2.32	0.87	0.90	0.53	0.61
Chm_7	163	124	469 (2.88)	0.56	2.38	0.90	0.88	0.53	0.62
Chm_8	110	71	300 (2.73)	0.55	2.30	1.86	0.90	0.53	0.60
Total	1233	886	429 (2.79)	0.55	2.35	1.01	0.90	0.54	0.62

TP, total primers; TNP, total number of polymorphic primers; Na, numbers of alleles; MF, major allelic frequency; Ne, number of effective alleles; *I*, Shannon's information index; *Ho*, observed heterozygosity; *He*, expected heterozygosity; PIC, polymorphic information content.

### Wet-lab validation through PCR

**Figure 4:**
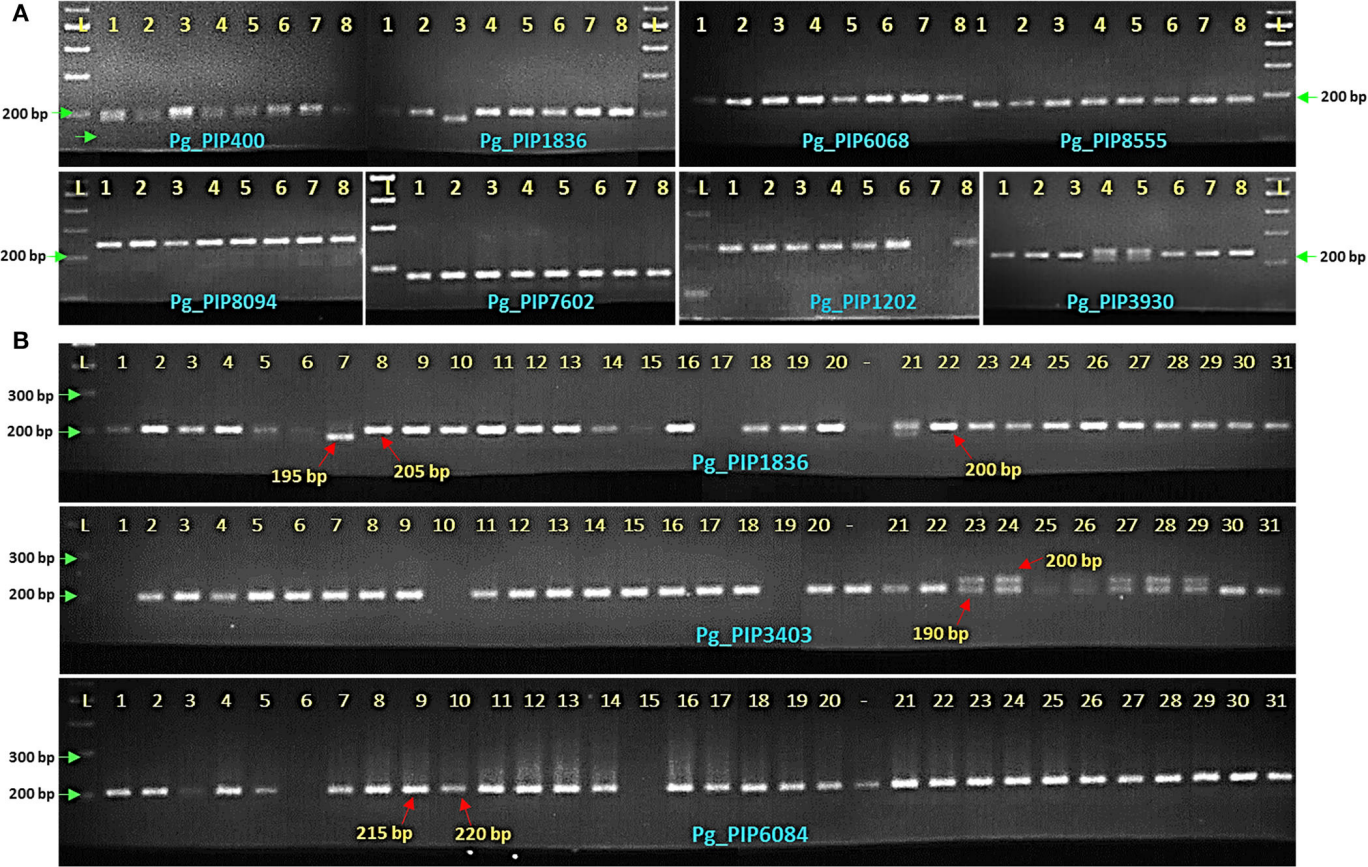
Allelic differences for PIP markers when validated on eight pomegranate genotypes **(A)**, 31 genotypes using PIP markers Pg_PIP1836, 3403, and 6084 on 3% metaphor gels **(B)** (Lane L-100 bp DNA ladder, lanes 1–8, eight genotypes as mentioned in the Section Materials and methods, lanes 1–31, genotypes as mentioned in Table 1, lane-, genotypes not considered for analysis).

For wet-lab validation, we synthesized a set of 100 PIP markers randomly distributed across chromosomes of Tunisia and screened on eight pomegranate genotypes (Supplementary Table 6). As a result, 99 (99%) PIP primers could produce gene-specific amplicons in test genotypes. Of these, 76 (76%) PIP markers revealed polymorphisms across eight pomegranate genotypes, 12 were monomorphic, and 11 markers showed amplifications in one to three genotypes but one marker did not show amplification. The representative gel profiles of pomegranate genotypes using selected PIP markers are shown in Figure 4. Using PIP markers, we detected 177 alleles among eight pomegranate genotypes, and PIC values varied from 0 to 0.53 with a mean value of 0.30 (Supplementary Table 7). It is worth noting that 15 PIP markers had PIC values ≥0.50.

### *In silico* comparative mapping of PIP markers between pomegranate genomes

**Figure 5:**
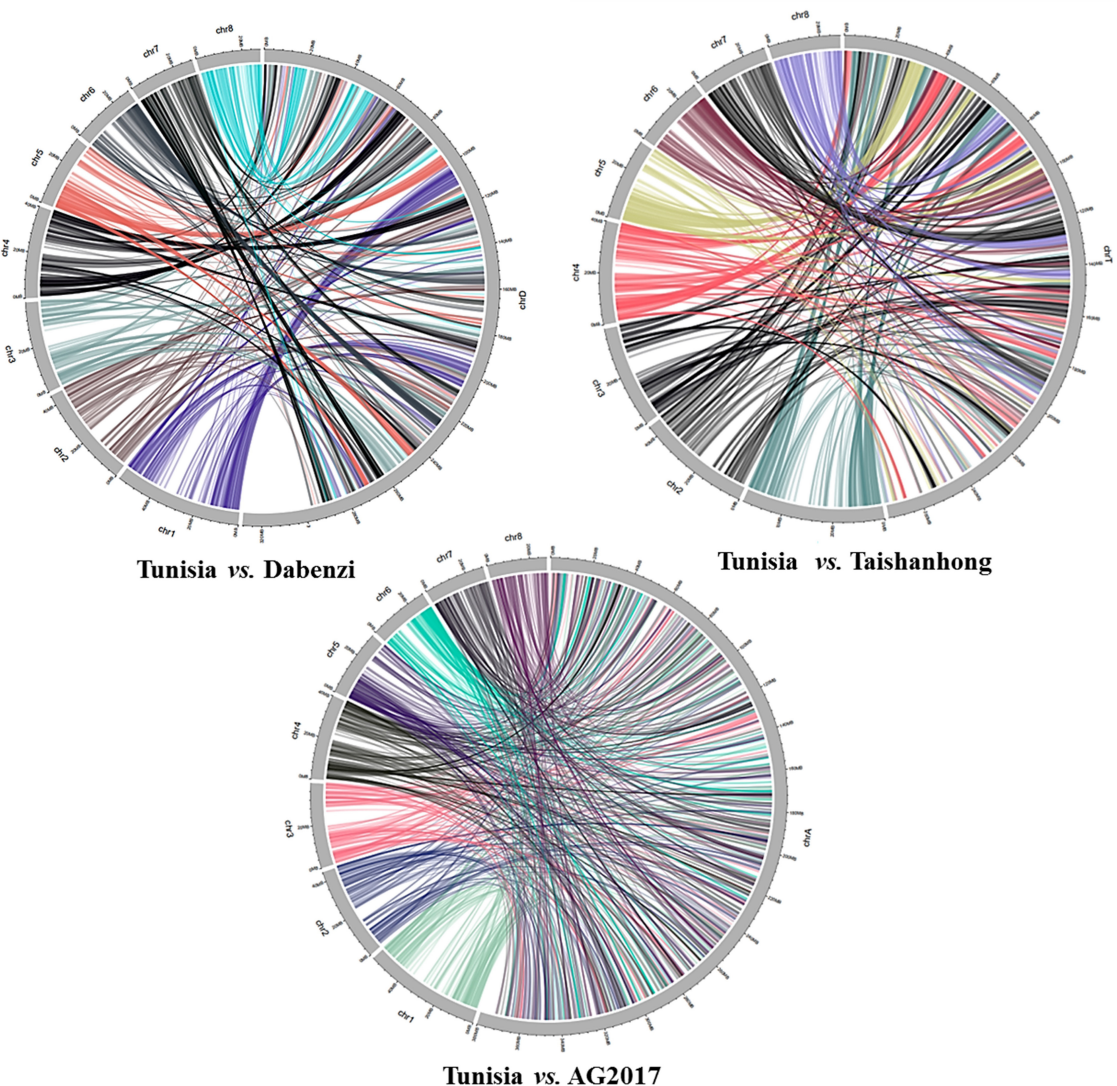
Syntenic relationships between Tunisia in comparison to Dabenzi, Taishanhong and AG2017 genomes based on 1233 PIP markers.

The physical locations of the 1,233 PIP markers mapped on the Tunisia genome were compared to their locations on three other draft genome assemblies (Figure 5). For markers spanning eight chromosomes, the results suggested the strongest syntenic relationship of Tunisia with Taishanhong (98.86%, 1,219) and Dabenzi (98.38%, 1,213), followed by AG2017 (94.97%, 1,171) (Table 4).

**Table 4:** A summary of 1233 PIP marker-based comparative mapping of Tunisia chromosomes with Dabenzi, Taishanhong, and AG 2017 assemblies, revealing syntenic relationships.

**Tunisia chromosomes**	**No of PIP markers**	**No of markers mapped on the assemblies**		
		**Dabenzi**	**Taishanhong**	**AG2017**
Chm_1	170	169 (99.41%)	167 (98.24%)	158 (92.94%)
Chm_2	120	117 (97.50%)	117 (97.50%)	116 (96.66%)
Chm_3	157	155 (98.73%)	157 (100%)	151 (96.18%)
Chm_4	234	228 (97.44%)	230 (98.29%)	224 (95.73%)
Chm_5	137	135 (98.54%)	136 (99.27%)	125 (91.24%)
Chm_6	142	140 (98.59%)	141 (99.30%)	135 (95.07%)
Chm_7	163	159 (97.55%)	161 (98.77%)	156 (95.71%)
Chm_8	110	110 (100%)	110 (100%)	106 (96.36%)
Total	1233	1213 (98.38%)	1219 (98.86%)	1171 (94.97%)

### Functional classification of PIP markers

**Figure 6:**
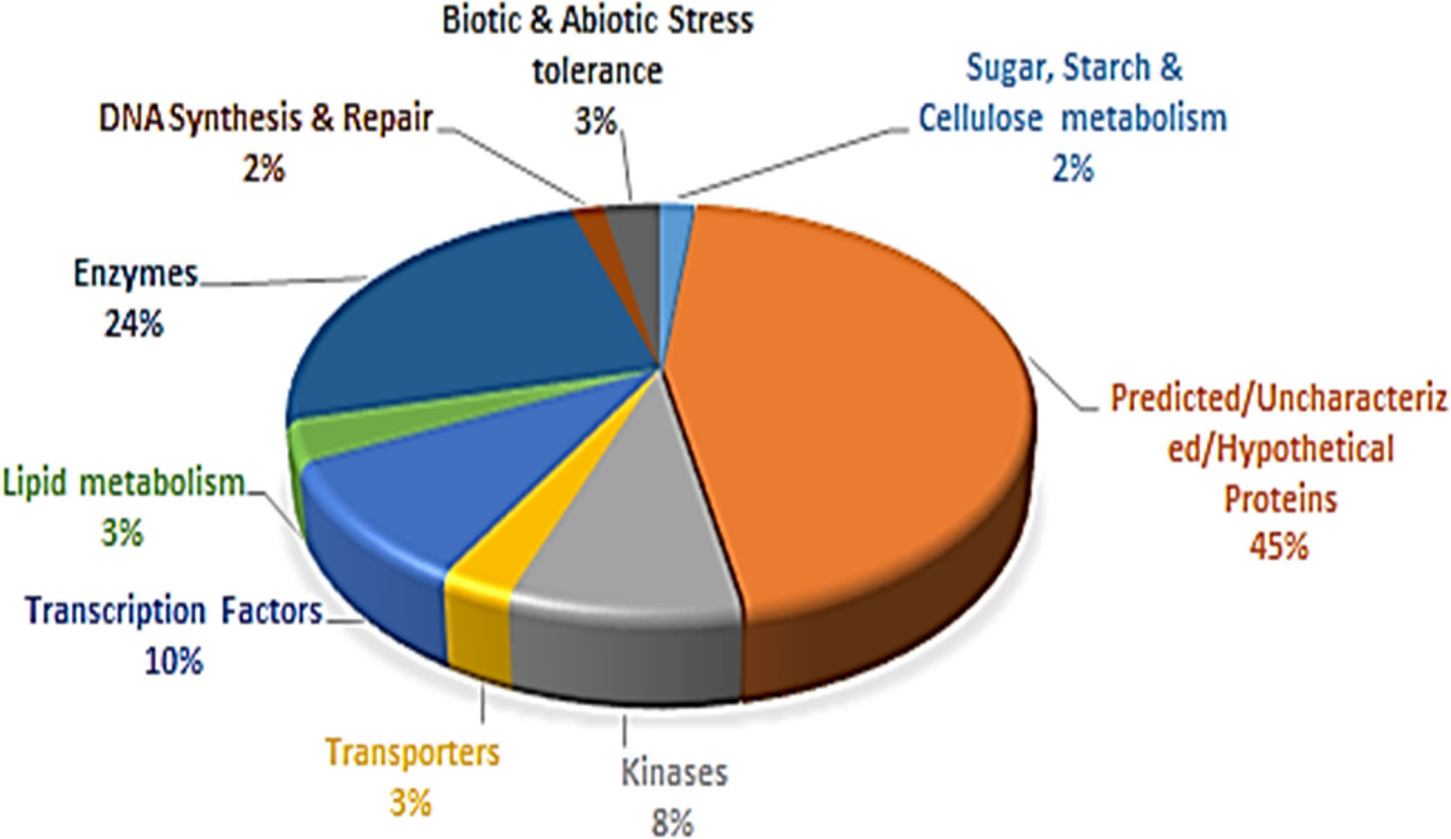
Proportionate distribution and classification of 3,445 annotated genes having 8,812 PIP markers.

Based on the available functional annotations of 3,445 gene models of Tunisia, 94% of the PIP markers had defined functions and 6% had uncharacterized proteins. All the annotated genes were grouped into nine major categories (Figure 6, Supplementary Table 8). The category with predicted/uncharacterized/hypothetical protein activities was the most dominant (45%) accompanied by enzymes (24%). The transcription factors (10%) ranked third, followed by kinases (8%), biotic and abiotic stress tolerance (3%), lipid metabolism (3%), DNA synthesis and repair (2%), and sugar, starch, and cellulose metabolisms (2%) (Figure 6).

### Gene ontology analysis of single locus PIP markers

**Figure 7:**
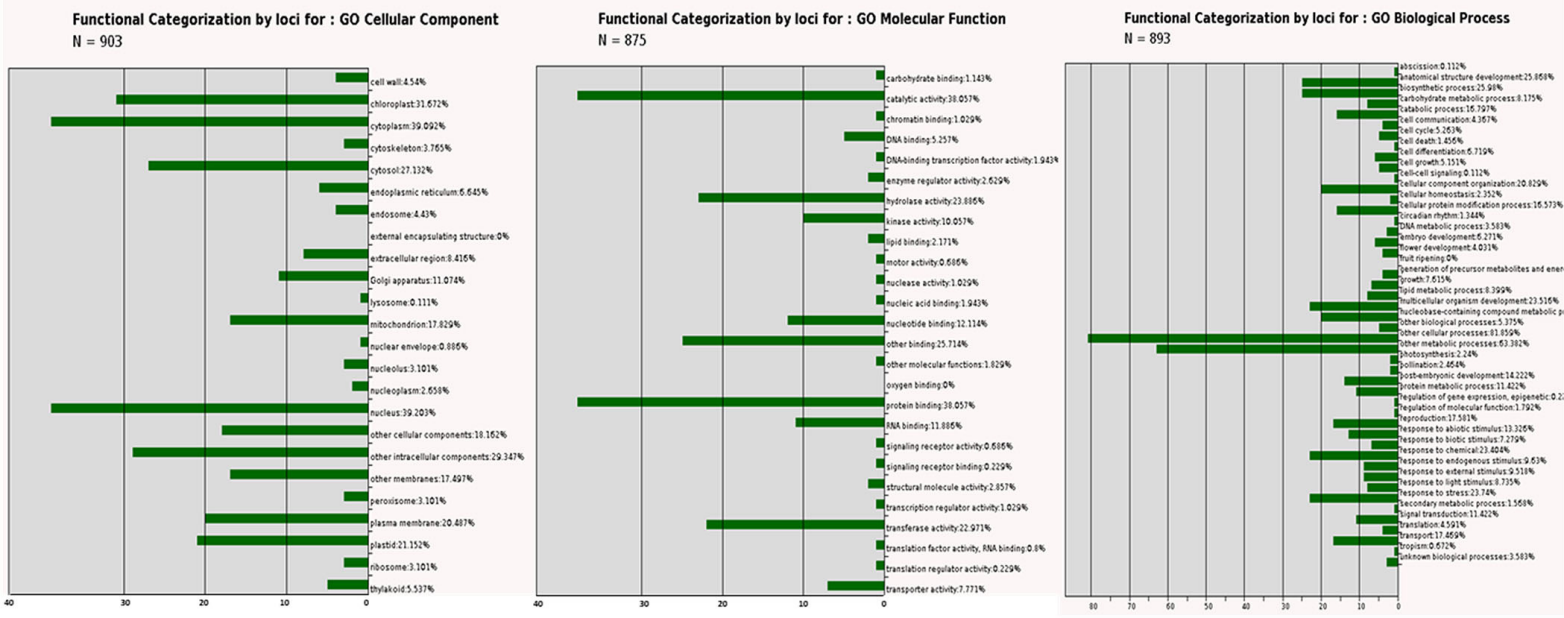
Gene ontology-based functional categorization of 958 genes with unique PIP marker amplifications in Tunisia genome that are part of (i) cellular component, (ii) molecular function, and (iii) biological process.

We performed gene ontology for 958 Arabidopsis homolog genes of pomegranate having single PIP marker amplicons to obtain GO Annotation Plot (Supplementary Table 9). All the genes were categorized into three classes: BP (47 GO terms), MF (26 GO terms), and CC (24 GO terms) (Figure 7). According to GO analysis, the majority of the genes under biological process were categorized into cellular processes (81.86%) and metabolic processes (63.38%). In CC, many genes are found in the nucleus (39%), the cytoplasm (39%), and the chloroplast (31.67%). However, in MF, many genes have a role in protein binding (38.06%), catalytic activity (38.06%), and other binding (25.71%).

### Genetic diversity

Based on the amplification and polymorphism profiles, a subset of 24 PIP markers distributed on 8 chromosomes of the Tunisia genome was selected for genotyping 31 pomegranate genotypes (Table 5). A total of 49 alleles were obtained across the genotypes, with an average value of 2.04 alleles. The ranges of expected heterozygosity and PIC were found as 0.28–0.49 and 0.28–0.50, respectively. Among the 31 pomegranate genotypes, the average “*I”* index was 0.59.

**Table 5:** Diversity parameters for 24 PIP markers screened on 31 pomegranate genotypes.

**Sl. no**	**Locus**	**Chm_location**	**N**	**Na**	**MF**	**Ne**	***I***	***He***	**PIC**
1	Pg_PIP207	Chr1	30	2	0.70	1.72	0.61	0.42	0.43
2	Pg_PIP259	Chr1	26	2	0.81	1.45	0.49	0.31	0.32
3	Pg_PIP400	Chr1	30	2	0.80	1.47	0.50	0.32	0.33
4	Pg_PIP1202	Chr1	31	2	0.71	1.70	0.60	0.41	0.42
5	Pg_PIP1312	Chr2	31	2	0.74	1.62	0.57	0.38	0.39
6	Pg_PIP1836	Chr2	30	3	0.82	1.46	0.58	0.31	0.32
7	Pg_PIP2740	Chr3	24	2	0.83	1.38	0.45	0.28	0.28
8	Pg_PIP2762	Chr3	31	2	0.71	1.70	0.60	0.41	0.42
9	Pg_PIP3008	Chr3	23	2	0.65	1.83	0.65	0.45	0.46
10	Pg_PIP3403	Chr4	31	2	0.63	1.88	0.66	0.47	0.47
11	Pg_PIP3549	Chr4	27	2	0.81	1.43	0.48	0.30	0.31
12	Pg_PIP3930	Chr4	28	2	0.57	1.96	0.68	0.49	0.50
13	Pg_PIP5131	Chr5	27	2	0.81	1.43	0.48	0.30	0.31
14	Pg_PIP6084	Chr6	30	2	0.73	1.64	0.58	0.39	0.40
15	Pg_PIP6525	Chr6	31	2	0.68	1.78	0.63	0.44	0.44
16	Pg_PIP6680	Chr6	30	2	0.77	1.56	0.54	0.36	0.36
17	Pg_PIP7532	Chr7	31	2	0.77	1.54	0.53	0.35	0.36
18	Pg_PIP7590	Chr7	21	2	0.57	1.96	0.68	0.49	0.50
19	Pg_PIP7602	Chr7	29	2	0.83	1.40	0.46	0.29	0.29
20	Pg_PIP8094	Chr8	28	2	0.57	1.96	0.68	0.49	0.50
21	Pg_PIP8194	Chr8	28	2	0.57	1.96	0.68	0.49	0.50
22	Pg_PIP8249	Chr8	29	2	0.62	1.89	0.66	0.47	0.48
23	Pg_PIP8555	Chr8	26	2	0.65	1.83	0.65	0.45	0.46
24	Pg_PIP8731	Chr8	30	2	0.70	1.72	0.61	0.42	0.43
	Mean		28.42	49 (2.04)	0.71	1.68	0.59	0.40	0.40

N, number of genotypes with amplification; Na, number of alleles; MF, major allelic frequency; Ne, number of effective alleles; *I*, Shannon's information index; *He*, expected heterozygosity; PIC, polymorphic information content.

**Figure 8:**
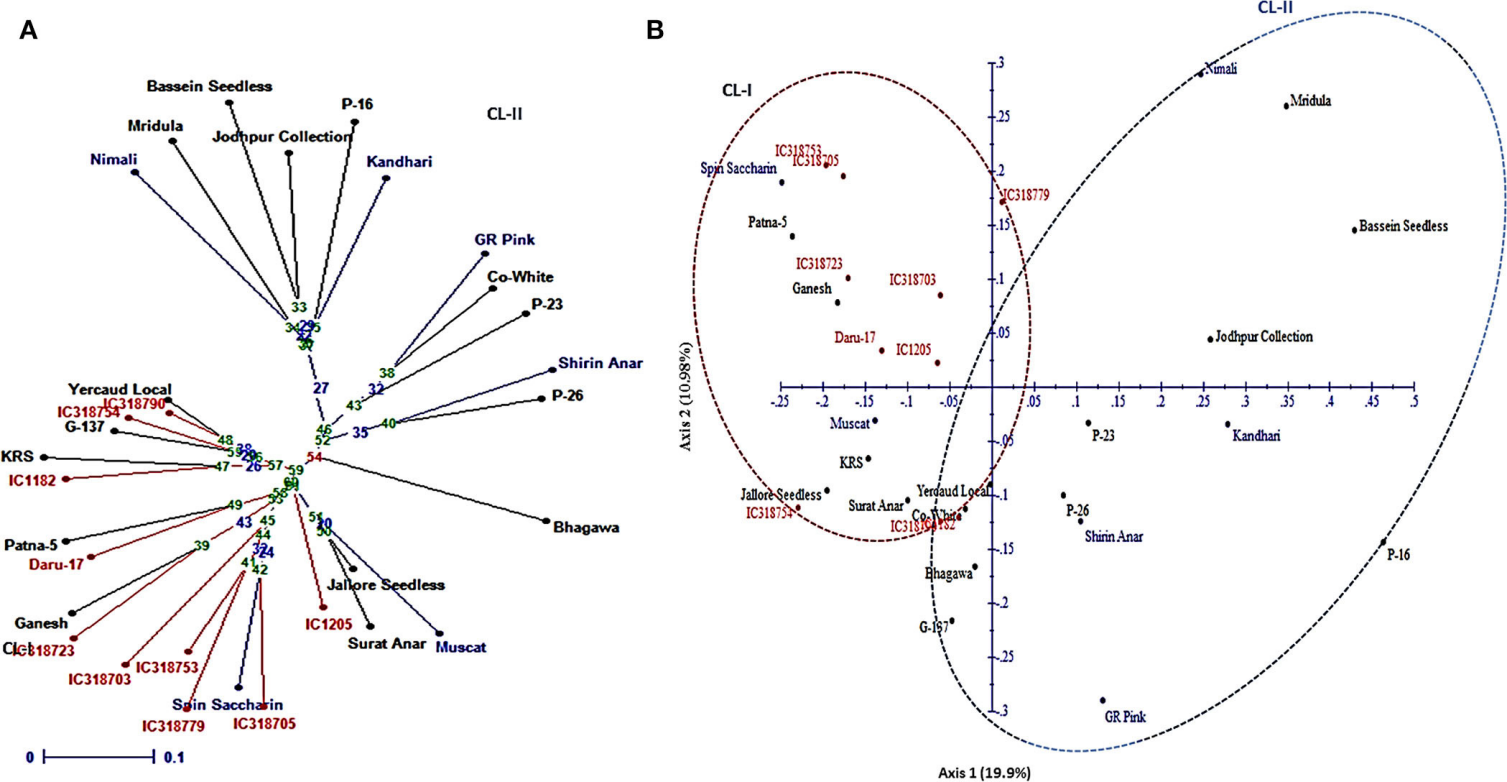
The Neighbor-joining tree **(A)** and the PCA plot **(B)** showing genetic relationships among 31 pomegranate genotypes based on 24 PIP markers.

Nineteen and 12 genotypes formed two separate clusters in the NJ tree based on 31 pomegranate genotypes (Figure 8A). Cluster 1 primarily had 10 wild genotypes, with an out-grouping of 9 cultivars. In addition, the PCA plot also separated 31 genotypes into two major groups (Figure 8B). The principal coordinates (PCos) 1 and 2 explained 19.9% and 10.98% of the total variance, respectively, accounting for 30.88% of the overall variation. Interestingly, PCo 1 distinguished two clusters into the wild and cultivar groups.

## Discussion

### Designing of PIP markers

In this study, we developed genome-wide PIP markers based on the information on individual introns obtained from the annotated gene models of the pomegranate genome (cv. Tunisia) using the PIP database. We designed 8,812 novel PIP markers targeting 3,445 gene models that spanned 8 chromosomes of the Tunisia genome. We found that the overall PIP marker density increased with shorter chromosome lengths. Wang et al. (2006) reported a considerable change in ILP density across the rice genome and among chromosomes. In pomegranate, we observed earlier a positive correlation between chromosomal length and SSR abundance, with higher marker density on the shorter chromosome length (Patil et al., 2021). Xia et al. (2017), while working on different marker systems (SSR, ILP, and PIP) in 16 tree species, discovered that genome size has a high correlation with the number of marker loci compared to marker density.

### Genome-wide distribution of PIP markers

As illustrated by the Circos graph, 8,812 PIP primers (targeted 3,445 genes) were scattered evenly across all of the 8 Tunisia chromosomes. In chickpea, 7,454 intron-spanning markers (ISMs) were developed from introns of 3,283 genes representing the entire eight chromosomes (Srivastava et al., 2016). Badoni et al. (2016) also developed 16,510 ILP markers, which were physically mapped on 12 chromosomes, and found them to be well-distributed throughout the rice genome. The development of multiple PIP markers from individual genes allows researchers to choose the best primer combination for reliable amplification.

### ePCR validation and high-density PIP marker-based physical map

The information on ILP markers was utilized to construct a high-density PIP marker-based physical map. Through *in silico* PCR, we identified 1,233 genome-wide Pg_PIP markers producing single amplicons in the Tunisia genome and physically mapped them to construct a PIP marker-based saturated physical map with an average marker density of 4.31 markers/Mb. Similarly, Muthamilarasan et al. (2014) reported the development of 5,123 ILP markers, 4,049 of which were physically mapped onto 9 chromosomes of foxtail millet with a marker density of 9.8 markers/Mb. The ePCR technique was found to be highly useful in the validation and identification of informative markers that are derived through *in silico* methods in many crops (Cui et al., 2017; Wang et al., 2018; Uncu and Uncu, 2020). Several studies demonstrated the use of a marker-based physical map to fine-map QTL regions (Zhao et al., 2017). In our previous research, we constructed a high-density physical map based on SSR loci, which could act as a reference for assessing genotyping data for different types of populations in pomegranate.

Although we designed primers based on the Tunisia gene models, we were able to validate 7,425 primers (84.26%) and failed to acquire e-PCR products for 1,387 (15.74%) primers in the Tunisia genome. This could be due to constraint conditions set while designing ILP primers and e-PCR validation that resulted in mismatches between primers with the genomic sequence as also observed in rice (Wang et al., 2006).

### Identification of polymorphic PIP markers

The most crucial characteristic of any molecular marker system is its ability to uncover a high level of DNA polymorphism. Therefore, we used *in silico* simulated PCR to determine the PIP marker polymorphism across four pomegranate genome sequences. As a result, 1,233 (Tunisia) to 1,283 (Taishanhong) markers showed single ePCR product with the expected size across the multiple genomes. With a mean PIC value of 0.62, 886 (71.86%) markers were found to be polymorphic, which implied their highly informative nature and would serve as a valuable genomic tool for downstream trait mapping in pomegranate. Similarly, Patil et al. (2021) identified the 265 most informative SSR markers with an average PIC value of 0.46 through ePCR validation of *in silico*-designed markers across multiple pomegranate genomes. Recently, Patil et al. (2022) reported the identification of 77 polymorphic miRNA-SSRs for seed hardness breeding through multiple genome-ePCR confirmations in pomegranate.

### *In silico* comparative genome mapping

We showed the utility of the PIP marker-based physical map to facilitate comparative genome mapping in pomegranate. A total of 1,233 physically mapped markers were compared with the three different pomegranate draft genomes. We noticed 98.38% (1,213) to 98.86% (1,219) markers mapped to Dabenzi and Taishanhong genome assemblies, respectively, with lower than 94.97% in AG2017 (1,171) genome. This revealed a considerable proportion of sequence-based orthology and syntenic relationship due to the cross-transferability nature of ILP markers within genera or cross-genera as that of SSR markers. These results also substantiated conserved orthologous genes that are evenly distributed across pomegranate genomes. Muthamilarasan et al. (2014) reported ~85% transferability for foxtail millet ILP markers within eight millets and five non-millet species.

The interspecies conservation of PIP markers renders these marker systems highly suitable for generating cross-species genetic markers (Huang et al., 2013). Our comparative mapping revealed the highest conserved syntenic blocks of Tunisia with Dabenzi and Taishanhong assemblies, whereas the least synteny was observed with AG2017. Xia et al. (2017) assessed SSR and ILM markers for duplication analysis in trees, and the syntenic graphs elucidated that SSRs corresponded to substantially higher duplication occurrences than gene-based ILS markers, implying the suitability of SSRs for duplication study in tree species.

### Functional annotation of PIP markers

The genome-wide ILP markers developed from a variety of cloned or functionally annotated candidate genes could facilitate gene-trait association studies and genomics-assisted breeding in different crop species (Badoni et al., 2016). Therefore, based on annotation details, 3,445 Tunisia gene models with PIP markers were grouped into nine categories. The largest category (45%) contained gene sequences with predicted/uncharacterized/hypothetical protein functions. Similarly, the largest category (47.4%) belonged to hypothetical/uncharacterized/putative functions when analyzed for 5,123 ILP markers in foxtail millet (Muthamilarasan et al., 2014). We found that the second largest category comprised enzymes (24%); pomegranate being a medicinal plant, there is every chance that investigating 45% of their functions (predicted/uncharacterized/hypothetical protein) could help to identify important unique enzymes that are part of potential biochemical pathways or structural proteins, which could help to improve fruit quality traits in future.

The gene ontology was analyzed for 958 Arabidopsis homolog genes having single PIP marker amplifications in pomegranate. All the genes were categorized into one of three GO categories: BP (47), MF (26), and CC (24). In biological processes, 81.86% of genes engaged in cellular processes, whereas 63.38% of genes engaged in metabolic processes. The majority of genes were engaged in protein binding, catalytic activity, and other binding activities in molecular functions. The bulk of genes was found in the nucleus, the cytoplasm, and the chloroplast. The relevance of these gene-derived PIP markers for pomegranate trait mapping was clearly demonstrated by GO analysis. Saminathan et al. (2016) performed large-scale sequencing and identification of ncRNAs during fruit development stages in pomegranate. Through GO for miRNA target genes, they found the majority of genes to possess binding and catalytic activity, having a role in cellular and metabolic processes, and are part of the cell, intracellular, and organelle parts. Similarly, Patil et al. (2020b) also performed the GO analysis for miRNA target genes to elucidate the importance of gene-derived miRNA-SSRs markers for trait mapping in pomegranate. In a recent study, we performed GO for 727 gene targets of miRNAs that are part of seed development (Patil et al., 2022) and observed biological processes to be the most abundant category. Binding and catalytic activity had the highest representation in the molecular functions category.

### Wet-lab validation of PIP markers

We performed wet lab validation experiments for 100 PIP markers, of which 76 markers were polymorphic on eight genotypes. The marker polymorphism data of ILP markers as assessed on metaphor gels enabled us to obtain an average PIC value of 0.30. Similar values of average PIC were earlier recorded for ILP markers in foxtail millet (0.20), cowpea (0.34), maize (0.48), and rice (0.45) (Wang et al., 2006; Gupta et al., 2012; Liu et al., 2012; Muthamilarasan et al., 2014). Following the criterion laid by Botstein et al. (1980), in our study, 15 PIP markers had PIC values ≥0.50, indicating the informative nature of these markers for genetic diversity and genetic mapping analyses in pomegranate. Similarly, Zhang et al. (2017) identified 25 ILP markers with PIC values >0.5 in *Medicago sativa* for genetic studies. In the rubber tree, Bhusudsawang and Ukoskit (2019) also found 20 ILP markers with PIC values >0.5. We observed lesser alleles with lower PIC values for PIP markers and many of the earlier reports in different crops clearly indicated ILP markers with higher alleles and PIC values when separated on acrylamide gels. This suggested limited resolution for PIP markers as that of SSRs when assayed on agarose and metaphor gels as compared to the high-end automated gel systems (Patil et al., 2020a). Therefore, the PIP markers generated here might show a higher degree of polymorphism on polyacrylamide or capillary systems.

### Genetic diversity based on PIP markers

The ability of selected PIP markers to detect polymorphism and molecular diversity among 31 pomegranate genotypes was evaluated by large-scale validation and genotyping. Recently, the potential of these novel markers to precisely assay large-scale genotyping, allelic diversity, and expression profiling in a diverse array of accessions was demonstrated in rice (Badoni et al., 2016).

There were 49 alleles with PIC values ranging from 0.28 to 0.50 and a mean value of 0.40 among the 24 PIP markers representing 8 chromosomes. PIP markers were found to be the best complement for SSR-based profiling in many other crops (Huang et al., 2013) and are codominant in nature with high cross-genera transferability. The new findings support our previous findings, in which we found 30 alleles for 13 HvSSRs originating from the “Dabenzi” genome with PIC values ranging from 0.12 to 0.63 across 46 genotypes (Patil et al., 2020a). Low PIC values for PIP markers were found in our investigation, which could be related to the low resolution of the agarose or metaphor gels employed for gel separation and scoring or to the smaller number of polymorphic alleles found in the cultivars investigated here, as in the SSR-based study (Patil et al., 2020a).

The mean Shannon's information index obtained for 24 PIP markers was 0.59. Our findings indicated that, among the 31 genotypes studied, there was a moderate level of genetic diversity. Patil et al. (2021) also found moderate genetic variability among 30 pomegranate genotypes using 16 chromosome-specific HvSSR markers. Similarly, the utility of gene-derived miRNA-based SSR markers for genetic diversity study was demonstrated in pomegranate in an earlier study (Patil et al., 2020b, 2022).

The NJ tree based on 24 PIP markers divided 31 genotypes into two separated groups constituting wild and cultivated types. These findings strongly corroborate the clustering patterns recorded previously in pomegranates using SSRs and miRNA-SSRs (Patil et al., 2020a,b, 2022). It was interesting to note that cluster 2 was found to be more diverse by including a few introduced exotic pomegranate accessions like Nimali (Srilanka), Kandhari (Afghanistan), GR Pink (Russia), and Shirin Anar (Russia). Similarly, Patil et al. (2021) observed the inclusion of exotic lines, which accounted for higher genetic diversity levels as observed for cultivars groups based on SSR markers. The PCA plot also grouped 31 genotypes into two major clusters as that of the NJ tree. The PCos 1 accounted for a higher proportion of the variance of 19.9%, separating wild type from cultivars in pomegranate as reported in previous studies (Patil et al., 2020a, 2022). Overall, the NJ tree and the factorial analysis showed strong agreements based on the PIP markers.

## Conclusion

The present study reports the development of novel gene-based PIP markers evenly distributed on eight Tunisia chromosomes. *In silico* survey of PIP markers in the 36,524 annotated gene models of the Tunisia genome resulted in the designing of 8,812 PIP markers specific to 3,445 gene models that spanned 8 chromosomes. Further, we first assessed the *in silico* amplification of all the PIP markers, identified 1,233 markers that amplified a single locus, and corresponded to 958 important genes of Tunisia. In this study, we provided a set of 886 polymorphic PIP markers after ePCR validation on four pomegranate genome assemblies. Furthermore, amplification of 100 PIP markers was confirmed through wet-lab experiments, with 76% of them being polymorphic. Comparative mapping of 1,233 PIP markers across four pomegranate genomes revealed a significant proportion of orthology and syntenic relationships of Tunisia with Dabenzi and Taishanhong assemblies, followed by AG2017. The immediate use of the developed PIP markers was exemplified by a genetic diversity study of 31 pomegranate genotypes. The study provides an important functional marker resource for future trait discovery and improvement and for genomics-assisted breeding of pomegranate.

## Data availability statement

The datasets presented in this study can be found in online repositories. The names of the repository/repositories and accession number(s) can be found in the article/Supplementary material.

## Author contributions

PP and RM were designed the research experiments. PP and SJ were performed *in silico* analyses and were carried out wet-lab experiments. MN and SP assisted in the collection of test materials. PP, AB, and AM wrote the original manuscript with the help of RM. All authors approved the final version to submit.

## Funding

This research was funded by the Indian Council of Agricultural Research (ICAR), New Delhi, India, through the ICAR-National Research Centre on Pomegranate, Solapur (MS), as part of an Institute Project.

## Conflict of interest

The authors declare that the research was conducted in the absence of any commercial or financial relationships that could be construed as a potential conflict of interest.

## Publisher's note

All claims expressed in this article are solely those of the authors and do not necessarily represent those of their affiliated organizations, or those of the publisher, the editors and the reviewers. Any product that may be evaluated in this article, or claim that may be made by its manufacturer, is not guaranteed or endorsed by the publisher.

## Supplementary material

Supplementary Table 1 Details for 8812 PIP primers designed from 3,445 genes of Tunisia genome.

Supplementary Table 2 Details of locations of genes and PIP markers on Tunisia chromosomes.

Supplementary Table 3 Physical position of PIP markers on 8 chromosomes of Tunisia.

Supplementary Table 4 Details of 1233 PIP primers designed from 958 genes of Tunisia genome.

Supplementary Table 5 Marker parameters for 1233 PIP primers assayed across four pomegranate genomes using ePCR.

Supplementary Table 6 Details of 100 PIP primers used for wet-lab validation among six pomegranate genotypes.

Supplementary Table 7 Marker statistics obtained based on 100 PIP primers screened on 8 pomegranate genotypes.

Supplementary Table 8 Functional categorization of 3,445 annotated Tunisia genes with 1,233 PIP markers.

Supplementary Table 9 Details of 956 Arabidopsis homolog genes of pomegranate having single PIP marker amplicons used for GO annotation.
