# Supplementary material for: Chromosome-specific potential intron polymorphism markers for large-scale genotyping applications in pomegranate
Source: Front Plant Sci. 2022 Aug 30;13:943959. doi: 10.3389/fpls.2022.943959 (PMC9468638; doi:10.3389/fpls.2022.943959)
Supplement: Supplementary Table 6 — Details of 100 PIP primers used for wet-lab validation among six pomegranate genotypes. [file Table_6.docx]

**Suppl Table S6**. Details of hundred PIP primers used for wet lab validation among six pomegranate genotypes

| **Markers Code** | **Chms location** | **Forward Primer (5'-3')** | **Reverse Primer (5'-3')** | **GC%** | **Tm** | **Intron length** | **Product size** | **Annotations** |
| --- | --- | --- | --- | --- | --- | --- | --- | --- |
| **Pg_PIP266** | **chr1** | CATTGTCCAGGAGATCAGTGG | ACCTTGCTCCCCTTGGTTAT | 51.2 | 60.2 | 90 | 199 | soluble inorganic pyrophosphatase 1-like |
| **Pg_PIP564** | **chr1** | GGCTCACCTGTTTCCTTGTT | CCATATCTTCTCGGGGATAAAA | 45.5 | 59.3 | 110 | 211 | ATP-dependent zinc metalloprotease FTSH 12, chloroplastic |
| **Pg_PIP1051** | **chr1** | CCAGGCTATCAACACCACAA | TGATCACAGTCCACGTTCAG | 50 | 58.9 | 79 | 183 | cellulose synthase A catalytic subunit 7 [UDP-forming] |
| **Pg_PIP260** | **chr1** | ACATTCAGTCTCGGGGTCGT | TTCACTATTGCGTGCATTCC | 50 | 60.8 | 90 | 200 | endoribonuclease Dicer homolog 1 |
| **Pg_PIP566** | **chr1** | CCTGTTGTGTCGGTTGATTG | AAGGGGATTCCTTTGCTCAT | 47.5 | 60 | 79 | 181 | thiosulfate/3-mercaptopyruvate sulfurtransferase 1, mitochondrial-like |
| **Pg_PIP1202** | **chr1** | CAACGGACATCTGCTGTTTT | TTCCAACTGATTTGAAAGTAGTCAA | 38.5 | 59 | 85 | 191 | probable manganese-transporting ATPase PDR2 |
| **Pg_PIP400** | **chr1** | GAGCAGCTCATTAGTGGCAAG | GGATCCTGTCCAAGCAGAGA | 53.7 | 60.3 | 93 | 193 | tubulin alpha chain-like |
| **Pg_PIP460** | **chr1** | TGTATAACACCATAGCTCGTGTGAC | TCGCAAAACATGGTCAGAAA | 42 | 60.3 | 85 | 186 | ATP-citrate synthase beta chain protein 2-like |
| **Pg_PIP259** | **chr1** | AGTCAAGAGATGCGGACGTG | TGTCGAATATCCAGCCCTTC | 52.5 | 60.7 | 88 | 201 | endoribonuclease Dicer homolog 1 |
| **Pg_PIP207** | **chr1** | GCACTCCATATTGGATGTCTCC | TCCTGTTGCCATCTCGATAA | 47.5 | 60 | 84 | 188 | mitogen-activated protein kinase kinase kinase NPK1-like |
| **Pg_PIP554** | **chr1** | CAAAGAAGATTCTGAAGGTGTGG | GGACTGCTCGTGAACATGC | 50.7 | 60.3 | 90 | 191 | UPF0613 protein PB24D3.06c-like |
| **Pg_PIP1642** | **chr2** | AACTCCTTCGGTGATGAGCA | GAAGCTCGGGCACCTCTT | 55.6 | 60.7 | 91 | 209 | sedoheptulose-1,7-bisphosphatase, chloroplastic |
| **Pg_PIP1859** | **chr2** | GAGTCACATGCCAAAGACCA | ATGCCAAACTCCTTCGCATA | 47.5 | 60.1 | 89 | 189 | protein argonaute 10 |
| **Pg_PIP1403** | **chr2** | CGAGATCATGAGGAATACCCTTT | CTCGAAGGCAAGAAGGTCAG | 49.2 | 60.4 | 100 | 212 | V-type proton ATPase subunit d2 |
| **Pg_PIP2272** | **chr2** | CTACGCCACCAGGTCCAAC | ATCCTCTTCCCGGTGACTG | 60.5 | 60.8 | 79 | 196 | 60S ribosomal protein L34 |
| **Pg_PIP1573** | **chr2** | CCATACACTCCCACCACCTT | GCATCTCACGGAAATGGTGT | 52.5 | 60.3 | 83 | 183 | NADH--cytochrome b5 reductase 1 |
| **Pg_PIP1814** | **chr2** | AGGTTCCCGATCCTGGTTAG | TGGAACATCTTCTTGTACTGCTTC | 48.3 | 60.1 | 90 | 197 | CLP protease regulatory subunit CLPX1, mitochondrial |
| **Pg_PIP1823** | **chr2** | TACTTACTCGAGCCGCTTTG | GAGGGGCGTCACCAACAC | 58.3 | 60.5 | 86 | 194 | probable magnesium transporter NIPA6 |
| **Pg_PIP1803** | **chr2** | TCCTGTGAACACAAGGGAGA | TGTCCCATGGACAACCAAAT | 47.5 | 60.1 | 93 | 202 | potassium channel AKT1-like |
| **Pg_PIP1836** | **chr2** | TGTCTCGCGTATGGAGATACT | ATGCAGGACCACCTGAATTT | 46.3 | 58.4 | 91 | 195 | protease Do-like 9 |
| **Pg_PIP1312** | **chr2** | TCGATGAGTTCGACAAAATGA | ACTGTAGTTATGCCGGCTTTG | 42.9 | 59.3 | 99 | 199 | DNA replication licensing factor MCM5 |
| **Pg_PIP1499** | **chr2** | CAGATTTCATATATGGGTTTTCCTG | AGATATGAGAGAACCGGAAAGG | 40.7 | 59.4 | 93 | 193 | probable UDP-N-acetylglucosamine--peptide N-acetylglucosaminyltransferase SEC |
| **Pg_PIP1341** | **chr2** | CATTCATCTTAAAGGGAACCAAA | TTCAGCTGTTGTATCCCCAGT | 41.2 | 59.5 | 75 | 184 | tRNase Z TRZ2, chloroplastic |
| **Pg_PIP1911** | **chr2** | GACGGAGTTTCATATACTCATTCTTG | GAGGAAGCCCTTCCAAGTTT | 44.2 | 59.6 | 91 | 192 | ADP-ribosylation factor-related protein 1 |
| **Pg_PIP2799** | **chr3** | TGTGGAGAAGCATGAATTTGTT | GTTGCTTTAGTCCTTTGGAAAA | 36.4 | 58.6 | 105 | 225 | kinesin-like protein KIN-13B |
| **Pg_PIP2614** | **chr3** | CGCCAAGTATGGAACTACTCATC | TGATCTGCTAAAGTTCCACCAA | 44.4 | 59.9 | 82 | 199 | NADP-dependent malic enzyme-like |
| **Pg_PIP2365** | **chr3** | TGATGTTTACAGTTTTGGAGTGG | CCAGCGGACCAAAGATTG | 47.3 | 59.6 | 95 | 195 | protein STRUBBELIG-RECEPTOR FAMILY 6 |
| **Pg_PIP2762** | **chr3** | CACGGGATTTCTGGACTCTG | AGAAGCTACAGCTGCGTTGG | 55 | 60.7 | 75 | 188 | homeobox-leucine zipper protein REVOLUTA |
| **Pg_PIP2348** | **chr3** | TCTGGTTTCGTCAGTTGGTG | GAAGTCTTCCCCCATCTTTTT | 46.4 | 59.1 | 87 | 187 | protein TIC 62, chloroplastic |
| **Pg_PIP3008** | **chr3** | GAGGGAGAAAATCAGTGAGAGG | CGTTGAAGTGATTGCACGTAG | 48.8 | 59.4 | 88 | 208 | transcription factor bHLH49 |
| **Pg_PIP3239** | **chr3** | TGACAAAGATCTACCACATGATTG | TTTCTGTGTCAGGCATTTTCC | 41.3 | 59.5 | 132 | 232 | nucleosome assembly protein 1;3-like |
| **Pg_PIP3235** | **chr3** | GCTCTCACGAGGTTTCAAGG | GTGATCTCGAGTGCCTCTGG | 57.5 | 60.5 | 81 | 192 | eukaryotic initiation factor 4A-10-like |
| **Pg_PIP2722** | **chr3** | AGGAGGATGATGTCAGGAAA | AATGGAGTCACCAGCCTCTG | 50 | 58.7 | 97 | 206 | 40S ribosomal protein S6 |
| **Pg_PIP2411** | **chr3** | TTTTGGTACTTCGGGAAAGG | CGTACACAAGCTTCAAGTATCCA | 44.2 | 59.2 | 96 | 197 | alanine--glyoxylate aminotransferase 2 homolog 1, mitochondrial |
| **Pg_PIP2479** | **chr3** | CTCGCCGAGAATATGATGG | GTCCGCGTGATCTTCAAAAG | 51.3 | 60 | 148 | 249 | serine/threonine-protein kinase SRK2A-like |
| **Pg_PIP3084** | **chr3** | AGCTCTTGGAGGTGTTGAGG | TTCAAAACCAGACGCCTTTT | 47.5 | 59.6 | 139 | 242 | pre-mRNA-processing-splicing factor 8A |
| **Pg_PIP2740** | **chr3** | GGCAATGACTTGTACAGGGAAA | GGGCTCATTCATTTGACCAT | 45.2 | 60.5 | 101 | 206 | ATP synthase subunit beta, mitochondrial-like |
| **Pg_PIP4106** | **chr4** | AGCAACTTATCTCGGGCAAG | CCTTACTCGGTCAAGGCAGA | 52.5 | 59.9 | 77 | 178 | tubulin alpha-3 chain-like |
| **Pg_PIP4149** | **chr4** | AGGGCAAGGTAGGAGAGGTT | GAGAGCATGACAGTGCTGGT | 55 | 59.1 | 163 | 283 | probable F-actin-capping protein subunit beta |
| **Pg_PIP3549** | **chr4** | AACGATGCTAGGCTTGCTCT | GGACGCCACTTGAGTAAAGAA | 48.8 | 59.3 | 80 | 183 | UDP-sugar pyrophosphorylase |
| **Pg_PIP3371** | **chr4** | TTCGAACCCTGACAAGTTCC | TGGCCCATATATCAGCAAGA | 47.5 | 59.6 | 79 | 184 | ras-related protein RABC1 |
| **Pg_PIP3959** | **chr4** | CAGGTCTTTGAAGGTGAAAGG | ACCTCAATCTGAGGGGTTCC | 51.3 | 59.6 | 106 | 213 | endoplasmic reticulum chaperone BiP |
| **Pg_PIP4638** | **chr4** | GTTGGCTCTGGTGGAGAAAG | TGCTTTTCGATATCAATTCCTG | 45.7 | 59.5 | 150 | 251 | monodehydroascorbate reductase, chloroplastic/mitochondrial |
| **Pg_PIP3930** | **chr4** | AACGGCACTTAACTTCATACCAA | TCATCAACTGCAGCTTCACC | 44.6 | 60 | 94 | 197 | phospholipase SGR2 |
| **Pg_PIP3627** | **chr4** | CATTGGTGGCACAAAAGTTG | GAGCATCAGAGCTCCAAACC | 50 | 60 | 100 | 212 | cytochrome c biogenesis protein CCS1, chloroplastic |
| **Pg_PIP4592** | **chr4** | GGAGATGTTGGAGCTGGAAA | CAGCCAACGTTTGAGAGAAG | 50 | 59.4 | 92 | 204 | ras-related protein RABF2b |
| **Pg_PIP4506** | **chr4** | TTCCGATCTCTTCTGGGTTG | ATTCCCGACAGCATGTGTAA | 47.5 | 59.6 | 85 | 185 | triose phosphate/phosphate translocator, chloroplastic |
| **Pg_PIP3639** | **chr4** | TCTCGAGATTGCCGAAGAAT | TTCATGTCGCTCTGTTCCAA | 45 | 60.2 | 104 | 204 | protein translocase subunit SECA1, chloroplastic |
| **Pg_PIP3849** | **chr4** | GCTTCATGGGATGGCTTTC | TCAATTTGATATATCCCTTTATACGC | 41.7 | 59.7 | 108 | 212 | E3 ubiquitin protein ligase RIN2-like |
| **Pg_PIP3403** | **chr4** | CTCTCTCATTGCCGGGATTA | TGCATACTCCGAATGAAATCTT | 43.2 | 59.4 | 86 | 194 | protein ORANGE, chloroplastic |
| **Pg_PIP5564** | **chr5** | AGATGATCGGACCATTGTCTTT | AGAAGCTCCGTGGAATGTCTA | 44.3 | 59.4 | 79 | 180 | thylakoid lumenal 15.0 kDa protein 2, chloroplastic |
| **Pg_PIP5645** | **chr5** | TCGTTGTTAGTGGTCGTGGT | TTGATTACTTTGGGAGGTCCA | 46.4 | 59.2 | 71 | 171 | putative elongation factor TypA-like SVR3, chloroplastic |
| **Pg_PIP5618** | **chr5** | TGCTTGCCTGTACATTGCTT | TTCTTTTGCTCGTCCTATTTCC | 43 | 59.6 | 87 | 196 | transcription initiation factor IIB-2 |
| **Pg_PIP5214** | **chr5** | AAATCGAAGGGGCATCAAT | TGCTCTTCAGATCTCGGTGT | 46.1 | 58.9 | 75 | 194 | probable LRR receptor-like serine/threonine-protein kinase At1g67720 |
| **Pg_PIP5360** | **chr5** | AGCAGTTGAGAAGGGGGAAT | TTGCCTGCTTACACACCAGT | 50 | 59.7 | 150 | 250 | valine--tRNA ligase, chloroplastic/mitochondrial 2 |
| **Pg_PIP5744** | **chr5** | CAAAACAAACTGGGGGTTG | GCTAGACTCCTCAGTTTGCTGAA | 47.6 | 59.5 | 151 | 258 | syntaxin-71 |
| **Pg_PIP5111** | **chr5** | GAACTCTTAACTGACGAGCCATT | TGACATGATCAAAGTCCACCA | 43.2 | 59.5 | 82 | 182 | peptidyl-prolyl cis-trans isomerase CYP65 |
| **Pg_PIP4918** | **chr5** | TGGAGAACTTGAGGAACATGG | AAAACGATCAGCCTTGACCA | 46.3 | 60.4 | 97 | 216 | 5'-nucleotidase domain-containing protein 4 |
| **Pg_PIP5131** | **chr5** | TTTCAGGAACTACTGCCAATG | GATAATTTCGACGCCAGGAA | 43.9 | 59 | 80 | 186 | chloride channel protein CLC-d |
| **Pg_PIP4939** | **chr5** | AGATGGAGCTGTTGGGAAAA | TCAACCATCACATTGGCACT | 45 | 59.8 | 85 | 196 | rac-like GTP-binding protein RAC2 |
| **Pg_PIP5760** | **chr5** | CGGCTAGCAAAGATCCTCAG | CATGAAAGCAAGGAGGGAAA | 50 | 60.1 | 76 | 178 | translin |
| **Pg_PIP5522** | **chr5** | TCATTGTCGATGGTTCTTGG | GCTAACAGTCGGTAAGCAAACA | 45.2 | 59.5 | 83 | 186 | protein CHROMATIN REMODELING 20 |
| **Pg_PIP5721** | **chr5** | TGGCCGATGAAGACTTAAAA | CTAAGCTTCCCCAACCACAC | 47.5 | 59 | 81 | 198 | tuberculostearic acid methyltransferase UfaA1-like |
| **Pg_PIP6134** | **chr6** | CTTCTTGGAGCTGACGGAAT | CAATGGCTTCCCTAATTTTCC | 46.4 | 59.6 | 88 | 201 | phototropin-1 |
| **Pg_PIP6818** | **chr6** | ACGACCCATTCGACATCTTC | AATGGATGAACCACATCTTCG | 46.4 | 59.9 | 93 | 199 | dnaJ protein homolog ANJ1 |
| **Pg_PIP6287** | **chr6** | TGGACATGGCCTAATGTCAAT | TGTACAATCTCTTGCTCATGTTCC | 42.3 | 60.4 | 79 | 181 | uncharacterized LOC116211146 |
| **Pg_PIP6680** | **chr6** | CTCCCTCGAATCAAGGAAAA | AGTTCACCCTGCACCTGAGT | 50 | 59.5 | 96 | 205 | protein PECTIC ARABINOGALACTAN SYNTHESIS-RELATED |
| **Pg_PIP6525** | **chr6** | GCTCTGGTCGCTCGTATGTT | GACCTGATCCTAGATCACTTCCA | 51.4 | 60 | 83 | 191 | outer envelope protein 80, chloroplastic |
| **Pg_PIP6084** | **chr6** | GGAGTGTCGGTGTCATGCTA | TCACTCGTGAAATCAATGTGG | 48.9 | 59.6 | 104 | 219 | calcium-dependent protein kinase 34-like |
| **Pg_PIP6442** | **chr6** | TTGCAGACCACTTGTGAGGA | CGTCTAAGAGCTGTGCCATC | 52.5 | 59.5 | 87 | 203 | AP-2 complex subunit alpha-1-like |
| **Pg_PIP5973** | **chr6** | TTTTCATCCTGGCATTTTCC | CAACAAGAATGATCGGCACA | 42.5 | 60.3 | 74 | 185 | rac-like GTP-binding protein ARAC1 |
| **Pg_PIP6384** | **chr6** | GTTGCCCGGTTCATACAAAA | CGTAATCGTAGCTGGTCGTG | 50 | 60.1 | 86 | 201 | beta-galactosidase 3 |
| **Pg_PIP6180** | **chr6** | GATCAATATTGGGGGAAGCA | AAATGGCTCATCAATTTGAGAAA | 37.7 | 59.8 | 118 | 222 | probable transmembrane GTPase FZO-like, chloroplastic |
| **Pg_PIP6058** | **chr6** | CGCTGTTAAAACCCTCAACC | CCAATTAGCTTAACCAAGTTAGGA | 43.8 | 58.8 | 82 | 190 | probable serine/threonine-protein kinase PIX7 |
| **Pg_PIP6052** | **chr6** | GGTGCTTCCTGTATATGTTTATGATG | AACACCCCAGCCCTCAAT | 47 | 60.2 | 101 | 210 | calpain-type cysteine protease DEK1 |
| **Pg_PIP5886** | **chr6** | TGAATGAGGCTAGCGACAGA | AATCCCTCCATCCCTTCAAT | 47.5 | 59.6 | 161 | 261 | uncharacterized LOC116210128 |
| **Pg_PIP7108** | **chr7** | TCATCCTTTGACGAGGACAA | CCATTACAGAATCTTTCATCTGGA | 41.3 | 59.4 | 84 | 191 | E3 ubiquitin-protein ligase RGLG2-like |
| **Pg_PIP7428** | **chr7** | CCTACAGGTTCCGGTTTCC | TCTTCGTCGCGGTGAAATA | 52.6 | 59.9 | 121 | 227 | protein PLASTID TRANSCRIPTIONALLY ACTIVE 10 |
| **Pg_PIP7590** | **chr7** | TCTGCCTGTACTTGCATTCG | ACCAGGATTTGCCTTCACAA | 47.5 | 60.3 | 110 | 210 | putative exosome complex component rrp40 |
| **Pg_PIP7647** | **chr7** | CTAGAGTGCACGCAGGAAAG | TGAGTTGAACCAGTTGTGATTT | 45.7 | 58 | 103 | 214 | 2-oxoglutarate and iron-dependent oxygenase JMJD4 |
| **Pg_PIP7340** | **chr7** | GTGTCATCCCGAACTTCCTC | GAATGGCTTCGGAATGTCCT | 52.5 | 60.4 | 88 | 198 | ABC transporter G family member 11-like |
| **Pg_PIP7144** | **chr7** | TCGGCTTTCTCTTTCTTGTCA | AGGTAAGCAGTCGCAGTTCC | 48.9 | 59.8 | 91 | 206 | probable metal-nicotianamine transporter YSL7 |
| **Pg_PIP7473** | **chr7** | CACCCATGGCAGATTTGTTA | CTGCAGCTCCAATTCTTTCC | 47.5 | 59.7 | 140 | 248 | peptidyl-prolyl cis-trans isomerase FKBP42 |
| **Pg_PIP7602** | **chr7** | GCAAGAACCTAGGCAAACCA | TGGCAATTGAGTAATAGTGTCTGTT | 43 | 59.7 | 91 | 192 | 26S proteasome non-ATPase regulatory subunit 14 homolog |
| **Pg_PIP7320** | **chr7** | TGATGTACACGTTGGGCATT | AAAGGGCTTCTTCTAGCTTTCC | 45.2 | 59.7 | 76 | 177 | transmembrane emp24 domain-containing protein p24beta2 |
| **Pg_PIP7232** | **chr7** | AGCAAGTTCTGTCCCTACAACA | AAGCAAAGCAACACCAGGTT | 45.2 | 59.3 | 107 | 209 | homogentisate 1,2-dioxygenase |
| **Pg_PIP7532** | **chr7** | TTCTTTGAGGTTGCTGTGGA | TCCCTTGTCACGTCTCTTGA | 47.5 | 59.4 | 80 | 180 | CCA-adding enzyme-like |
| **Pg_PIP8754** | **chr8** | GGGCATCTCCTGCATTCTT | ATTCGTCGTTTTCGACGTG | 50 | 59.9 | 114 | 214 | omega-3 fatty acid desaturase, chloroplastic-like |
| **Pg_PIP8137** | **chr8** | GGCAGTGATGGCTAAAGCTAA | GTTCGGCCATAGAATGGTTT | 46.3 | 59.2 | 113 | 214 | NADH dehydrogenase [ubiquinone] 1 alpha subcomplex subunit 9, mitochondrial |
| **Pg_PIP8731** | **chr8** | ATATATTGCTGTGGTGCTGGAA | ATTCACGGCCGGTATGGTA | 46.8 | 60.3 | 132 | 232 | proteasome subunit beta type-7-A-like |
| **Pg_PIP8249** | **chr8** | ATGGTAGGAAGCATAACCACAA | CAGTCATGGACGGAACTCGT | 48 | 59.8 | 134 | 234 | WD repeat and HMG-box DNA-binding protein 1 |
| **Pg_PIP8748** | **chr8** | TTCCCTGGGTCATTATTGGA | CACAAGCTATGACCTTCAAACC | 45.2 | 59.5 | 94 | 211 | triosephosphate isomerase, cytosolic |
| **Pg_PIP8555** | **chr8** | AGCTACCTTACGGGAAACAGG | AAGAGTTCGGAAGAGGAAAAGG | 48.9 | 59.9 | 102 | 202 | protein ACTIVITY OF BC1 COMPLEX KINASE 1, chloroplastic |
| **Pg_PIP8556** | **chr8** | AGTAGATGAATTTGGCGAAAAG | AATTTTAACAGTGGGATCATCTTTA | 32.2 | 57.5 | 107 | 216 | protein ACTIVITY OF BC1 COMPLEX KINASE 1, chloroplastic |
| **Pg_PIP7867** | **chr8** | CATTTAATCGCAGACGGACA | GCAAAGAACTCCTAAGCTCCAA | 45.2 | 59.9 | 82 | 195 | serine/threonine-protein kinase Aurora-1-like |
| **Pg_PIP8093** | **chr8** | TGTGGACACACCATTCACAA | TTTTCTCCCATACCTTGGACA | 43.9 | 59.4 | 88 | 200 | probable E3 ubiquitin-protein ligase RZFP34 |
| **Pg_PIP8094** | **chr8** | TCTGAAACTTCCCGAGAACC | CCGCTGCAGTTTGTACAGATA | 48.8 | 59.1 | 100 | 202 | probable ADP-ribosylation factor GTPase-activating protein AGD14 |
| **Pg_PIP8194** | **chr8** | AATCCTCATGCCGATCTCC | ATTTGCATCATCCGCTCTTC | 48.8 | 60.1 | 88 | 204 | CHD3-type chromatin-remodeling factor PICKLE |
| **Pg_PIP8484** | **chr8** | TCACACAAACGGACTTGACA | ATACTGAGGCCTGCAACCTT | 47.5 | 58.7 | 92 | 197 | lysine--tRNA ligase, cytoplasmic-like |
| **Pg_PIP8343** | **chr8** | CCTTATCTTGGGATTTGTTTGG | CCGGATCAAATTCATTGCTA | 40.5 | 59.1 | 84 | 184 | CTP synthase |
