# Supplementary material for: Chromosome-specific potential intron polymorphism markers for large-scale genotyping applications in pomegranate
Source: Front Plant Sci. 2022 Aug 30;13:943959. doi: 10.3389/fpls.2022.943959 (PMC9468638; doi:10.3389/fpls.2022.943959)
Supplement: Supplementary Table 7 — Marker statistics obtained based on 100 PIP primers screened on 8 pomegranate genotypes. [file Table_7.docx]

Suppl Table S7. Marker statistics obtained based on hundred PIP primers screened on 8 pomegranate genotypes

| **Sl.No** | **Markers Code** | **Chm**  **Location** | **N** | **Na** | **Ne** | **I** | **He** | **PIC** |
| --- | --- | --- | --- | --- | --- | --- | --- | --- |
| **1** | Pg_PIP266 | **chr1** | 2 | 1 | 1.00 | 0.00 | 0.00 | 0.00 |
| **2** | Pg_PIP564 | **chr1** | 7 | 2 | 1.69 | 0.60 | 0.41 | 0.44 |
| **3** | Pg_PIP1051 | **chr1** | 7 | 2 | 1.69 | 0.60 | 0.41 | 0.44 |
| **4** | Pg_PIP260 | **chr1** | 6 | 2 | 1.80 | 0.64 | 0.44 | 0.48 |
| **5** | Pg_PIP566 | **chr1** | 6 | 2 | 1.80 | 0.64 | 0.44 | 0.48 |
| **6** | Pg_PIP1202 | **chr1** | 6 | 2 | 1.80 | 0.64 | 0.44 | 0.48 |
| **7** | Pg_PIP400 | **chr1** | 8 | 2 | 1.60 | 0.56 | 0.38 | 0.40 |
| **8** | Pg_PIP460 | **chr1** | 8 | 2 | 1.28 | 0.38 | 0.22 | 0.23 |
| **9** | Pg_PIP259 | **chr1** | 8 | 2 | 1.88 | 0.66 | 0.47 | 0.50 |
| **10** | Pg_PIP207 | **chr1** | 7 | 2 | 1.69 | 0.60 | 0.41 | 0.44 |
| **11** | Pg_PIP554 | **chr1** | 5 | 2 | 1.92 | 0.67 | 0.48 | 0.53 |
| **12** | Pg_PIP1642 | **chr2** | 7 | 1 | 1.00 | 0.00 | 0.00 | 0.00 |
| **13** | Pg_PIP1859 | **chr2** | 8 | 2 | 1.88 | 0.66 | 0.47 | 0.50 |
| **14** | Pg_PIP1403 | **chr2** | 4 | 2 | 1.60 | 0.56 | 0.38 | 0.43 |
| **15** | Pg_PIP2272 | **chr2** | 5 | 2 | 1.47 | 0.50 | 0.32 | 0.36 |
| **16** | Pg_PIP1573 | **chr2** | 8 | 2 | 1.60 | 0.56 | 0.38 | 0.40 |
| **17** | Pg_PIP1814 | **chr2** | 6 | 2 | 1.80 | 0.64 | 0.44 | 0.48 |
| **18** | Pg_PIP1823 | **chr2** | 8 | 1 | 1.00 | 0.00 | 0.00 | 0.00 |
| **19** | Pg_PIP1803 | **chr2** | 8 | 2 | 1.88 | 0.66 | 0.47 | 0.50 |
| **20** | Pg_PIP1836 | **chr2** | 6 | 2 | 1.38 | 0.45 | 0.28 | 0.30 |
| **21** | Pg_PIP1312 | **chr2** | 6 | 2 | 1.80 | 0.64 | 0.44 | 0.48 |
| **22** | Pg_PIP1499 | **chr2** | 8 | 1 | 1.00 | 0.00 | 0.00 | 0.00 |
| **23** | Pg_PIP1341 | **chr2** | 8 | 2 | 1.28 | 0.38 | 0.22 | 0.23 |
| **24** | Pg_PIP1911 | **chr2** | 3 | 2 | 1.80 | 0.64 | 0.44 | 0.53 |
| **25** | Pg_PIP2799 | **chr3** | 7 | 2 | 1.32 | 0.41 | 0.24 | 0.26 |
| **26** | Pg_PIP2614 | **chr3** | 8 | 2 | 1.28 | 0.38 | 0.22 | 0.23 |
| **27** | Pg_PIP2365 | **chr3** | 7 | 2 | 1.32 | 0.41 | 0.24 | 0.26 |
| **28** | Pg_PIP2762 | **chr3** | 8 | 2 | 1.60 | 0.56 | 0.38 | 0.40 |
| **29** | Pg_PIP2348 | **chr3** | 7 | 1 | 1.00 | 0.00 | 0.00 | 0.00 |
| **30** | Pg_PIP3008 | **chr3** | 8 | 2 | 1.60 | 0.56 | 0.38 | 0.40 |
| **31** | Pg_PIP3239 | **chr3** | 8 | 2 | 1.60 | 0.56 | 0.38 | 0.40 |
| **32** | Pg_PIP3235 | **chr3** | 8 | 2 | 1.28 | 0.38 | 0.22 | 0.23 |
| **33** | Pg_PIP2722 | **chr3** | 8 | 1 | 1.00 | 0.00 | 0.00 | 0.00 |
| **34** | Pg_PIP2411 | **chr3** | 8 | 2 | 1.28 | 0.38 | 0.22 | 0.23 |
| **35** | Pg_PIP2479 | **chr3** | 8 | 2 | 1.28 | 0.38 | 0.22 | 0.23 |
| **36** | Pg_PIP3084 | **chr3** | 3 | 1 | 1.00 | 0.00 | 0.00 | 0.00 |
| **37** | Pg_PIP2740 | **chr3** | 6 | 2 | 1.80 | 0.64 | 0.44 | 0.48 |
| **38** | Pg_PIP4106 | **chr4** | 8 | 2 | 1.60 | 0.56 | 0.38 | 0.40 |
| **39** | Pg_PIP4149 | **chr4** | 8 | 1 | 1.00 | 0.00 | 0.00 | 0.00 |
| **40** | Pg_PIP3549 | **chr4** | 8 | 2 | 1.88 | 0.66 | 0.47 | 0.50 |
| **41** | Pg_PIP3371 | **chr4** | 8 | 2 | 1.28 | 0.38 | 0.22 | 0.23 |
| **42** | Pg_PIP3959 | **chr4** | 8 | 2 | 1.28 | 0.38 | 0.22 | 0.23 |
| **43** | Pg_PIP4638 | **chr4** | 1 | 1 | 1.00 | 0.00 | 0.00 | 0.00 |
| **44** | Pg_PIP3930 | **chr4** | 7 | 2 | 1.69 | 0.60 | 0.41 | 0.44 |
| **45** | Pg_PIP3627 | **chr4** | 8 | 2 | 1.60 | 0.56 | 0.38 | 0.40 |
| **46** | Pg_PIP4592 | **chr4** | 8 | 2 | 1.60 | 0.56 | 0.38 | 0.40 |
| **47** | Pg_PIP4506 | **chr4** | 7 | 1 | 1.00 | 0.00 | 0.00 | 0.00 |
| **48** | Pg_PIP3639 | **chr4** | 8 | 2 | 2.00 | 0.69 | 0.50 | 0.53 |
| **49** | Pg_PIP3849 | **chr4** | 6 | 2 | 1.38 | 0.45 | 0.28 | 0.30 |
| **50** | Pg_PIP3403 | **chr4** | 8 | 2 | 1.60 | 0.56 | 0.38 | 0.40 |
| **51** | Pg_PIP5564 | **chr5** | 7 | 2 | 1.32 | 0.41 | 0.24 | 0.26 |
| **52** | Pg_PIP5645 | **chr5** | 8 | 2 | 1.28 | 0.38 | 0.22 | 0.23 |
| **53** | Pg_PIP5618 | **chr5** | 7 | 2 | 1.69 | 0.60 | 0.41 | 0.44 |
| **54** | Pg_PIP5214 | **chr5** | 7 | 2 | 1.32 | 0.41 | 0.24 | 0.26 |
| **55** | Pg_PIP5360 | **chr5** | 5 | 2 | 1.92 | 0.67 | 0.48 | 0.53 |
| **56** | Pg_PIP5744 | **chr5** | 4 | 2 | 1.60 | 0.56 | 0.38 | 0.43 |
| **57** | Pg_PIP5111 | **chr5** | 4 | 2 | 1.60 | 0.56 | 0.38 | 0.43 |
| **58** | Pg_PIP4918 | **chr5** | 2 | 1 | 1.00 | 0.00 | 0.00 | 0.00 |
| **59** | Pg_PIP5131 | **chr5** | 8 | 2 | 1.60 | 0.56 | 0.38 | 0.40 |
| **60** | Pg_PIP4939 | **chr5** | 7 | 2 | 1.69 | 0.60 | 0.41 | 0.44 |
| **61** | Pg_PIP5760 | **chr5** | 7 | 2 | 1.69 | 0.60 | 0.41 | 0.44 |
| **62** | Pg_PIP5522 | **chr5** | 5 | 2 | 1.47 | 0.50 | 0.32 | 0.36 |
| **63** | Pg_PIP5721 | **chr5** | 8 | 2 | 1.28 | 0.38 | 0.22 | 0.23 |
| **64** | Pg_PIP6134 | **chr6** | 2 | 1 | 1.00 | 0.00 | 0.00 | 0.00 |
| **65** | Pg_PIP6818 | **chr6** | 8 | 1 | 1.00 | 0.00 | 0.00 | 0.00 |
| **66** | Pg_PIP6287 | **chr6** | 3 | 1 | 1.00 | 0.00 | 0.00 | 0.00 |
| **67** | Pg_PIP6680 | **chr6** | 8 | 2 | 1.60 | 0.56 | 0.38 | 0.40 |
| **68** | Pg_PIP6525 | **chr6** | 8 | 2 | 1.28 | 0.38 | 0.22 | 0.23 |
| **69** | Pg_PIP6084 | **chr6** | 8 | 2 | 1.88 | 0.66 | 0.47 | 0.50 |
| **70** | Pg_PIP6442 | **chr6** | 8 | 2 | 1.28 | 0.38 | 0.22 | 0.23 |
| **71** | Pg_PIP5973 | **chr6** | 1 | 1 | 1.00 | 0.00 | 0.00 | 0.00 |
| **72** | Pg_PIP6384 | **chr6** | 6 | 2 | 1.80 | 0.64 | 0.44 | 0.48 |
| **73** | Pg_PIP6180 | **chr6** | 1 | 1 | 1.00 | 0.00 | 0.00 | 0.00 |
| **74** | Pg_PIP6058 | **chr6** | 7 | 2 | 1.32 | 0.41 | 0.24 | 0.26 |
| **75** | Pg_PIP6052 | **chr6** | 8 | 2 | 1.28 | 0.38 | 0.22 | 0.23 |
| **76** | Pg_PIP5886 | **chr6** | 5 | 2 | 1.47 | 0.50 | 0.32 | 0.36 |
| **77** | Pg_PIP7108 | **chr7** | 5 | 2 | 1.92 | 0.67 | 0.48 | 0.53 |
| **78** | Pg_PIP7428 | **chr7** | 7 | 2 | 1.32 | 0.41 | 0.24 | 0.26 |
| **79** | Pg_PIP7590 | **chr7** | 5 | 2 | 1.92 | 0.67 | 0.48 | 0.53 |
| **80** | Pg_PIP7647 | **chr7** | 6 | 2 | 1.38 | 0.45 | 0.28 | 0.30 |
| **81** | Pg_PIP7340 | **chr7** | 8 | 2 | 1.88 | 0.66 | 0.47 | 0.50 |
| **82** | Pg_PIP7144 | **chr7** | 8 | 2 | 1.88 | 0.66 | 0.47 | 0.50 |
| **83** | Pg_PIP7473 | **chr7** | 8 | 1 | 1.00 | 0.00 | 0.00 | 0.00 |
| **84** | Pg_PIP7602 | **chr7** | 8 | 2 | 1.60 | 0.56 | 0.38 | 0.40 |
| **85** | Pg_PIP7320 | **chr7** | 8 | 1 | 1.00 | 0.00 | 0.00 | 0.00 |
| **86** | Pg_PIP7232 | **chr7** | 8 | 2 | 1.60 | 0.56 | 0.38 | 0.40 |
| **87** | Pg_PIP7532 | **chr7** | 8 | 2 | 1.60 | 0.56 | 0.38 | 0.40 |
| **88** | Pg_PIP8754 | **chr8** | 0 | 0 | 0.00 | 0.00 | 0.00 | 0.00 |
| **89** | Pg_PIP8137 | **chr8** | 8 | 2 | 1.28 | 0.38 | 0.22 | 0.23 |
| **90** | Pg_PIP8731 | **chr8** | 7 | 2 | 1.96 | 0.68 | 0.49 | 0.53 |
| **91** | Pg_PIP8249 | **chr8** | 8 | 2 | 1.60 | 0.56 | 0.38 | 0.40 |
| **92** | Pg_PIP8748 | **chr8** | 7 | 2 | 1.69 | 0.60 | 0.41 | 0.44 |
| **93** | Pg_PIP8555 | **chr8** | 8 | 2 | 1.60 | 0.56 | 0.38 | 0.40 |
| **94** | Pg_PIP8556 | **chr8** | 6 | 2 | 1.80 | 0.64 | 0.44 | 0.48 |
| **95** | Pg_PIP7867 | **chr8** | 8 | 1 | 1.00 | 0.00 | 0.00 | 0.00 |
| **96** | Pg_PIP8093 | **chr8** | 8 | 1 | 1.00 | 0.00 | 0.00 | 0.00 |
| **97** | Pg_PIP8094 | **chr8** | 8 | 2 | 1.88 | 0.66 | 0.47 | 0.50 |
| **98** | Pg_PIP8194 | **chr8** | 8 | 2 | 1.60 | 0.56 | 0.38 | 0.40 |
| **99** | Pg_PIP8484 | **chr8** | 8 | 2 | 1.60 | 0.56 | 0.38 | 0.40 |
| **100** | Pg_PIP8343 | **chr8** | 5 | 1 | 1.00 | 0.00 | 0.00 | 0.00 |
|  | Total / Mean |  | **6.62** | 177 | **1.45** | **0.42** | **0.28** | **0.30** |
|  |  |  |  | **1.77** |  |  |  |  |
